# Comparative genomic analysis of *Flavobacteriaceae*: insights into carbohydrate metabolism, gliding motility and secondary metabolite biosynthesis

**DOI:** 10.1186/s12864-020-06971-7

**Published:** 2020-08-20

**Authors:** Asimenia Gavriilidou, Johanna Gutleben, Dennis Versluis, Francesca Forgiarini, Mark W. J. van Passel, Colin J. Ingham, Hauke Smidt, Detmer Sipkema

**Affiliations:** 1 Laboratory of Microbiology, Wageningen University & Research, Stippeneng 4, 6708 WE Wageningen, The Netherlands; 2 Present address: Ministry of Health, Welfare and Sport, Parnassusplein 5, 2511 VX, The Hague, The Netherlands; 3 Hoekmine BV, Utrecht, The Netherlands

**Keywords:** Comparative genomics, *Flavobacteriaceae*, Marine, Host-associated, Free-living

## Abstract

**Background:**

Members of the bacterial family *Flavobacteriaceae* are widely distributed in the marine environment and often found associated with algae, fish, detritus or marine invertebrates. Yet, little is known about the characteristics that drive their ubiquity in diverse ecological niches. Here, we provide an overview of functional traits common to taxonomically diverse members of the family *Flavobacteriaceae* from different environmental sources, with a focus on the Marine clade. We include seven newly sequenced marine sponge-derived strains that were also tested for gliding motility and antimicrobial activity.

**Results:**

Comparative genomics revealed that genome similarities appeared to be correlated to 16S rRNA gene- and genome-based phylogeny, while differences were mostly associated with nutrient acquisition, such as carbohydrate metabolism and gliding motility. The high frequency and diversity of genes encoding polymer-degrading enzymes, often arranged in polysaccharide utilization loci (PULs), support the capacity of marine *Flavobacteriaceae* to utilize diverse carbon sources. Homologs of gliding proteins were widespread among all studied *Flavobacteriaceae* in contrast to members of other phyla, highlighting the particular presence of this feature within the *Bacteroidetes*. Notably, not all bacteria predicted to glide formed spreading colonies. Genome mining uncovered a diverse secondary metabolite biosynthesis arsenal of *Flavobacteriaceae* with high prevalence of gene clusters encoding pathways for the production of antimicrobial, antioxidant and cytotoxic compounds. Antimicrobial activity tests showed, however, that the phenotype differed from the genome-derived predictions for the seven tested strains.

**Conclusions:**

Our study elucidates the functional repertoire of marine *Flavobacteriaceae* and highlights the need to combine genomic and experimental data while using the appropriate stimuli to unlock their uncharted metabolic potential.

## Background

The family *Flavobacteriaceae* is the largest family of the *Bacteroidetes* phylum, and its members thrive in a wide variety of habitats. These Gram-negative, non-spore forming, rod-shaped, aerobic bacteria are commonly referred to as flavobacteria [1, 2]. The taxonomy of members of the family *Flavobacteriaceae* is considered controversial [3], with many members that have been renamed and uprooted several times since the first classification [1, 4]. For the past two decades, the family structure has been relatively stable, even though changes in the taxonomy are still occurring due to the large number of new isolates [1]. There has been a vast increase in the number of described genera within the *Flavobacteriaceae* from ten [5] to 158 [6] in the past 20 years. Due to this large number of members, the family has been divided into the following clades: Marine, *Capnocytophaga*, *Flavobacterium*, *Tenacibaculum-Polaribacter* and *Chryseobacterium*, based on 16S ribosomal RNA (rRNA) gene-based phylogenetic analysis [1]. Recently the genus *Chryseobacterium* has been reclassified and moved into the family *Weeksellaceae* [7]. *Flavobacteriaceae* are common in terrestrial and freshwater environments and in many cases are numerically predominant in marine habitats [8]. Within the *Flavobacteriaceae*, bacteria isolated from marine sources are widespread throughout all clades, but a large proportion belongs to the Marine clade [1]. To date, marine flavobacteria have been found either free-living or attached to detritus in the water column [9, 10]. Their lifestyle includes colonization of the surface of algae [11], but also close association with invertebrate animals such as sponges [12], corals [13] and echinoderms [14].

A large number of marine flavobacteria degrade high molecular weight macromolecules, such as complex polysaccharides and proteins, contributing to the carbon turnover in marine environments [9, 11, 15]. Earlier comparative genomics analyses showed that genomes of marine flavobacteria encode a relatively large number of both glycosyl hydrolases (GHs) and peptidases compared to other marine bacteria. Moreover, a similar number of peptidases was found as compared to other proteolytic specialists, suggesting the important role of flavobacteria in the degradation of both complex carbohydrates and proteins [16]. The capacity of flavobacteria to use macromolecules varies considerably, which is reflected by differences in a broad spectrum of enzymes known as carbohydrate-active enzymes (CAZymes) for the whole family. Genes that encode the protein machinery for polysaccharide binding, hydrolysis and transport are often organised in distinct polysaccharide utilization loci (PULs) [17] that appear unique for the *Bacteroidetes* phylum. The first described PUL was the starch utilization system (Sus) of the human gut symbiont *Bacteroides thetaiotaomicron* [18]. PULs in other saccharolytic *Bacteroidetes* encode proteins homologous to SusC and SusD. The SusC-like proteins act as TonB-dependent receptor transporters while the SusD-like proteins are carbohydrate-binding lipoproteins [19]. Within PULs, genes encoding these homologs can be found in close proximity to genes coding for CAZymes, such as GHs, polysaccharide lyases (PLs), carbohydrate esterases (CEs) and carbohydrate binding modules (CBMs). Several previous studies have indicated that sequenced genomes of marine *Flavobacteriaceae* feature high proportions of CAZymes and PULs [9, 11, 15, 16, 20, 21], reinforcing their adaptations towards biopolymer degradation.

Besides their specialization as degraders of complex organic matter, many members of the *Bacteroidetes,* and particularly *Flavobacteriaceae*, share another distinct feature, known as ‘gliding motility’ [22]. Whilst gliding has been described in bacteria that belong to different taxa, such as *Chloroflexi*, *Cyanobacteria*, *Proteobacteria* and *Bacteroidetes* [23, 24], this term has been used loosely and covers multiple molecular mechanisms. *Bacteroidetes* species use their own unique motility machinery that results in rapid gliding movement by pivoting, flipping or crawling over solid surfaces without the aid of flagella or pili [22, 25]. Gliding motility can be observed both microscopically by cells moving on glass slides, as well as on agar by colony spreading [1, 22]. Cell movement is driven by a molecular motor that is composed of several groups of proteins (Gld, Spr and Rem) and powered by the proton motive force [26–28]. Recent studies revealed that this form of motility involves the rapid movement of adhesins delivered to the cell surface by a novel secretion system, the type 9 secretion system (T9SS), which is highly conserved among *Bacteroidetes* species [29] and unrelated to other known bacterial secretion systems [22, 24, 26, 30]. This system has been extensively studied in the motile aquatic or soil-derived *Flavobacterium johnsoniae* and the non-motile human oral pathogen *Porphyromonas gingivalis* [31]. In *F. johnsoniae*, 27 proteins are involved in gliding motility and protein secretion (Additional file 3). Orthologs of the *F. johnsoniae* proteins involved in type 9 secretion are also found in *P. gingivalis* [32, 33]. They are common within [22], but apparently limited to members of the *Bacteroidetes* [33]. Nevertheless, the exact nature of the gliding motility mechanism and its relationship with the T9SS remain under debate.

Gliding motility has previously been linked to the production of secondary metabolites likely due to their common purpose (predation/defence) [23]. Several antibiotics (β-lactams, quinolones) have been previously isolated from *Flavobacteriaceae* strains [34–37], with some of them acting against recalcitrant targets such as methicillin-resistant *Staphylococcus aureus* [38, 39]. Other bioactive molecules derived from flavobacteria include cell growth-promoting [40] and antitumor [41] compounds, von Willebrand factor receptor antagonists [42], protease [43] and topoisomerase [44] inhibitors, as well as antioxidative and neuroprotective myxols [45]. Even though numerous intriguing molecules are flavobacterial products, the metabolic potential of the family has been poorly investigated. Most of the microbially derived bioactive molecules belong to polyketides, non-ribosomal peptides, saccharides, alkaloids or terpenes and are related through common, highly conserved biosynthetic gene clusters (BGCs) [46, 47]. Computational detection of these BGCs and structural prediction of their products allow microbial genomes to be mined for metabolites [46]. Further exploration of the secondary metabolism of marine flavobacteria might therefore unlock a vast resource of novel bioactive compounds.

Here, we performed a comparative genomics analysis to investigate characteristic biological features, such as the complex carbohydrate metabolism and gliding motility mechanism of the *Flavobacteriaceae* family, with a focus on the ‘understudied’ Marine clade [1]. In order to examine the relatedness underlying these properties with other bacterial groups that are abundant and equally important in the marine environment we included genomes of microorganisms belonging to *Cyanobacteria* and *Proteobacteria* phyla. Together with *Bacteroidetes*, they represent the most significant fraction of the global marine bacterioplankton [8, 48, 49]. Moreover, to discern traits that highlight niche-adaptation the same analysis was conducted, comparing host-associated and non-host associated flavobacteria. Of the many recently identified members of the Marine clade of the *Flavobacteriaceae*, only a few have been studied in detail beyond their isolation and initial physiological characterization. In this study, we determined the individual genome sequences of seven presumably novel flavobacteria isolated from marine sponges [50, 51] and associated their genomic content with phenotypic features in terms of their gliding motility and antimicrobial activity. Finally, in silico genome mining for BGCs was performed to elucidate the secondary metabolic potential of bacteria belonging to dominant marine phyla (*Cyanobacteria* and *Proteobacteria*) and particularly, to members of the *Flavobacteriaceae*.

## Results

### Genome properties and phylogeny

To determine the genomic characteristics of the seven *Flavobacteriaceae* isolates recently obtained from the sponges *Aplysina aerophoba* and *Dysidea avara* and to allow for comparison with publicly available genomes from other members of the *Flavobacteriaceae*, their genomes were elucidated using Illumina sequencing. Genome sizes of the seven strains ranged from 3.7 to 5.3 Mbp with an average GC content of 38% (Table 1). The assembly generated genomes with 13–83 contigs and N50 ranging from 0.1 to 1.3 Mbp. Estimated completeness was more than 98%, and redundancy was lower than 2% for all assembled genomes. Coverage per base was above 200x for all draft genomes, except for DN50 for which it was 77x. Total gene count varied between 3317 and 4738, while the percentage of coding sequences (CDS) in all the assembled genomes was more than 98%. On average, more than 75% of the total genes could be assigned to protein families (Pfams).

**Table 1:** Genome properties and quality metrics of the strains sequenced in this study

	Aa_C5	Aa_D4	Aa_F7	Da_B9	DN50	DN105	DN112
Size (Mbp)	3.7	3.7	4.2	4.3	5.3	4.8	4.7
Contigs	45	13	43	19	38	83	19
%GC	37	37	41	45	39	36	31
N50 (Mbp)	0.8	0.7	0.6	0.6	0.3	0.1	1.3
Per base coverage (x)	1331	357	298	248	77	207	261
Completeness (%)	98.7	98.7	99.3	99.7	99.7	99.7	99.3
Contamination	0.1	0.1	0.2	0.7	1.8	0.6	1.0
Total gene count	3317	3306	3776	4079	4738	4331	4069
CDS genes (%)	98.7	98.7	98.8	98.7	98.7	98.8	98.0
Number of 16S rRNA genes	1	1	2	1	3	1	1
Number of tRNA genes	36	36	37	45	50	40	63
Genes in Pfams (%)	80.9	80.9	77.8	74.8	70.2	73.2	77.8
Isolation source	*Aplysina aerophoba*	*Aplysina aerophoba*	*Aplysina aerophoba*	*Dysidea avara*	*Aplysina aerophoba*	*Aplysina aerophoba*	*Aplysina aerophoba*

**Fig. 1:**
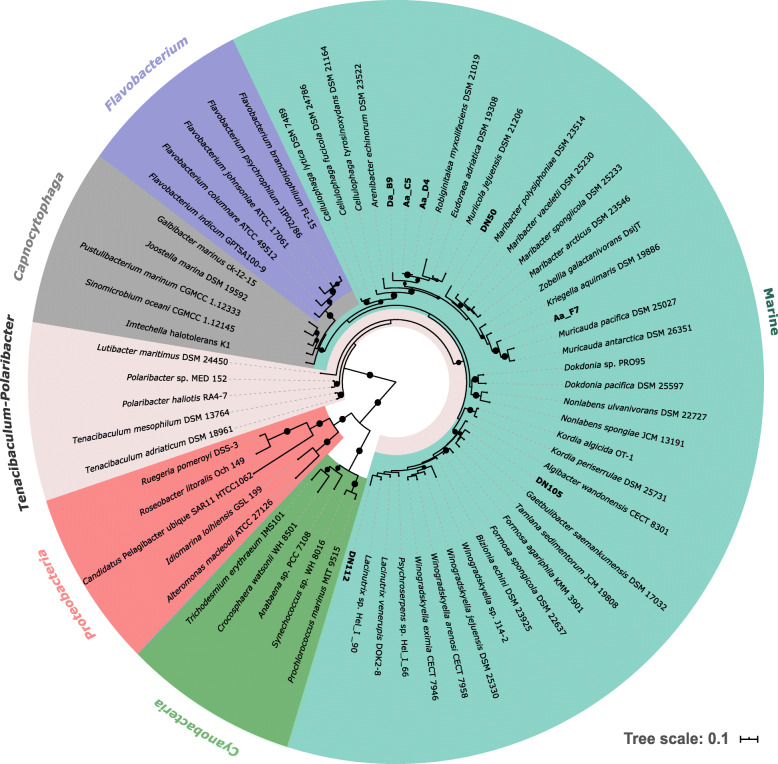
16S rRNA gene phylogenetic tree of microorganisms whose genomes were compared in this study. Phylogeny was inferred using a maximum likelihood method. Bootstrap values (> 70%) are displayed as black circles on the middle of the branches. Colour annotations represent the different *Flavobacteriacea*e clades. Sequences belonging to *Cyanobacteria* and *Proteobacteria* were used as outgroups. Names in bold indicate sequences generated in the current study. The scale bar indicates 0.1 substitutions per site

Phylogenetic analyses based on 16S rRNA gene as well as concatenated protein sequences both clustered all seven newly sequenced strains within the Marine clade of the family *Flavobacteriaceae* (Fig. 1 and Additional file 1; Fig. S1). All strains isolated in this study formed distinct branches in both phylogenetic trees, except for Aa_D4 and Aa_C5, the 16S rRNA gene sequences of which were 99.6% identical. Similarly, a two-way Amino Acid Identity (AAI) comparison of Aa_D4 and Aa_C5 resulted in 99.8% AAI. Among the newly sequenced isolates, two subclades within the Marine clade were represented, where DN112 and DN105 were phylogenetically adjacent, but distant from the rest of the isolates that grouped together (Fig. 1 and Additional file 1; Fig. S1). A BLASTN search against the nr/nt NCBI database showed that both Aa_C5 and Aa_D4 were members of the genus *Eudoraea* (Additional file 1; Table S2). BLASTN returned *Flagellimonas* sp. as the closest hit to Aa_F7 and *Lacinutrix* sp. to DN112. The closest related sequence to DN50 belonged to an uncultured organism clone, while Da_B9 and DN105 were most closely related to a *Flavobacteriaceae* bacterium, isolated from seamounts and a marine sponge, respectively (Additional file 1; Table S2). Taxonomic assignment of the newly sequenced genomes based on the Genome Taxonomy Database (GTDB) showed similar results with the 16S rRNA gene-based taxonomy. According to GTDB taxonomy, none of the strains could be classified to species level. Four of them were classified to genus level and the rest to family level (Additional file 1; Table S2).

### Functional profiling

**Fig. 2:**
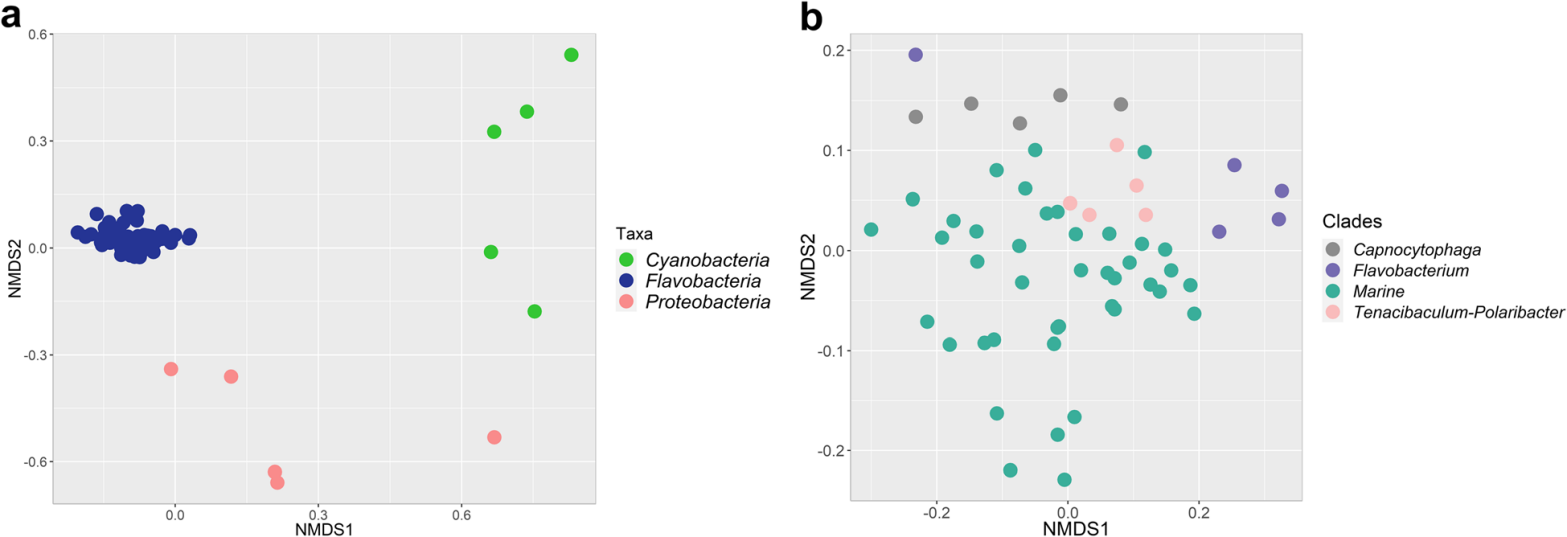
NMDS clustering of genomes based on similarity of functional groups according to Pfam annotations. Genomes are plotted following Bray-Curtis (dis) similarity values calculated from Pfam abundance profiles between major taxonomic groups (**a**) and different *Flavobacteriaceae* clades (**b**)

Pfam profiles of 66 genomes representing three taxonomic groups (the family *Flavobacteriaceae*, and the phyla *Cyanobacteria* and *Proteobacteria*), all four clades of the *Flavobacteriaceae* (Marine, *Capnocytophaga*, *Tenacibaculum*-*Polaribacter*, *Flavobacterium*) and two different types of isolation sources (host-associated and non-host associated) were used as input to assess the correlation of taxonomy and life strategy with genome-derived functional traits. *Flavobacteriaceae*, *Cyanobacteria* and *Proteobacteria* had highly divergent functional profiles based on the relative abundances of Pfam annotations (PERMANOVA, *p =* 0.001) (Fig. 2a). *Flavobacteriaceae* and *Cyanobacteria* showed higher overall dissimilarity (57.4%) at the functional level, compared to *Flavobacteriaceae* and *Proteobacteria* (50%). Similarly, functional profiles were significantly different between the four different flavobacterial clades (PERMANOVA, *p* = 0.001) (Fig. 2b). In contrast, no significant differences were observed between host-associated and non-host associated flavobacteria at the functional level (PERMANOVA, *p* > 0.05) (data not shown).

**Fig. 3:**
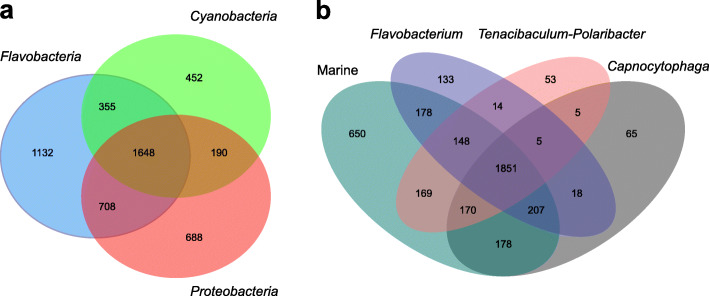
Shared and unique protein families (Pfams) between the analysed genomes. Venn diagrams illustrate shared and unique Pfams amongst *Flavobacteriaceae*, *Cyanobacteria* and *Proteobacteria* (**a**) and within the *Flavobacteriaceae* (**b**)

From the 5173 Pfams detected, 1648 were present in all three groups (*Flavobacteriaceae*, *Cyanobacteria* and *Proteobacteria*), whereas 1132 were specific to *Flavobacteriaceae* (Fig. 3a). The total number of predicted Pfams in the included genomes of the *Flavobacteriaceae* was 3843 with 17% of them being specific to the Marine clade. Overall, a high degree of functional conservation, was observed at the Pfam-level among the different clades of the *Flavobacteriaceae*, with 48% of all annotated Pfams being shared by the four clades (Fig. 3b). All predicted Pfams that significantly contributed most to the dissimilarity between *Flavobacteriaceae*, *Cyanobacteria* and *Proteobacteria* (SIMPER analysis, > 0.2% contribution, *p* <  0.05) showed higher abundances in *Flavobacteriaceae*. Particularly, most functional attributes strongly selected for in flavobacteria were related to proteins involved in carbohydrate metabolism and transport (Table 2). These include a series of protein domains found in TonB-dependent receptors (pfam13715, pfam07715, pfam00593) and two-component regulatory systems (pfam00072, pfam08281, pfam04542, pfam04397). The majority of the genes coding for TonB-dependent receptors were found adjacent to genes that code for SusD/RagB family proteins (pfam07980), which are restricted to the phylum *Bacteroidetes*. Moreover, among the most differentiating Pfam entries was the CHU_C family (pfam13585) that was found only in *Flavobacteriaceae*. This family has been reported as essential for the localization of a cellulase on the cell surface in *Cytophaga hutchinsonii* and the function of this cellulase in crystalline cellulose degradation [52]. It showed high similarity to the gliding motility-associated C-terminal domain (CTD) (TIGR04131) that is unique to and highly prevalent in the phylum *Bacteroidetes* [32]. This CTD (type B CTD) has been recently shown to target proteins for secretion by the T9SS [53]. Similarly, within the *Flavobacteriaceae*, the functional differences between the Marine, the *Capnocytophaga* and the *Flavobacterium* clade were mainly due to contribution of Pfam entries related to the attachment and degradation of polymeric compounds (Additional file 1; Table S3). There were no obvious functions distinguishing the Marine and the *Tenacibaculum*-*Polaribacter* clades, except for the C-terminal domain of the CHU protein family (pfam13585) that was significantly more abundant in the Marine clade members.

**Table 2:** Pfams contributing most significantly (> 0.2%, *p* <  0.05) to differences across major taxonomic groups

Function name (Pfam ID)	Relative Abundance (%)			Contribution (%)		*p*-values (< 0.05)	
	F	C	P	F-C	F-P	F-C	F-P
CarboxypepD_reg-like domain (pfam13715)	1.23	0	0.004	0.97	0.97	0.001	0.001
TonB-dependent Receptor Plug Domain (pfam07715)	1.13	0.01	0.44	0.77	0.76	0.001	0.016
TonB dependent receptor (pfam00593)	0.55	0.01	0.29	0.47	0.42	0.001	0.04
Response regulator receiver domain (pfam00072)	1.09	0.85	0.95	0.41	0.49	0.014	0.015
Sigma-70, region 4 (pfam08281)	0.51	0.04	0.11	0.41	0.37	0.001	0.002
LytTr DNA-binding domain (pfam04397)	0.40	0	0.06	0.35	0.34	0.005	0.029
Sigma-70 region 2 (pfam04542)	0.63	0.24	0.21	0.33	0.40	0.004	0.001
C-terminal domain of CHU protein family (pfam13585)	0.38	0	0	0.31	0.35	0.001	0.001

F *Flavobacteriaceae*, C* Cyanobacteria*, P* Proteobacteria*

### Carbohydrate metabolism and transport

**Fig. 4:**
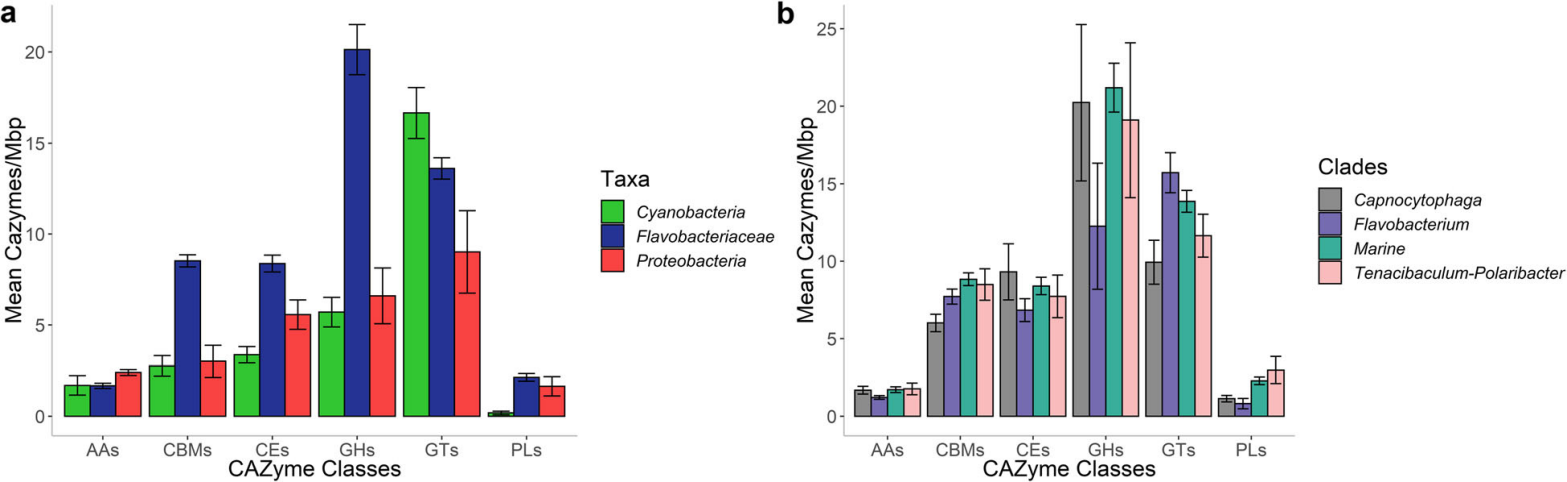
Mean number of CAZymes per Mb in the analysed genomes. Frequency of CAZymes normalized by total gene counts is shown between different taxonomic groups (**a**) and *Flavobacteriaceae* clades (**b**). Error bars represent mean numbers ± standard error. GHs, glycoside hydrolases; PLs, polysaccharide lyases; CEs, carbohydrate esterases; GTs, glycosyl transferases; CBMs, carbohydrate-binding modules

The ability to degrade and transform complex carbohydrates was assessed by mining the genomic data for carbohydrate-active enzymes (CAZymes) and PULs. In total, *Flavobacteriaceae* harboured more CAZymes per Mbp (Kruskal-Wallis test, *p* < 0.001) compared to *Cyanobacteria* and *Proteobacteria* (Additional file 4). Comparison of the CAZyme repertoire (average number of CAZymes per Mbp) across the different groups showed significantly higher frequencies of GHs, PLs, CEs and CBMs in *Flavobacteriaceae* (Kruskal-Wallis test, *p* < 0.001), whereas glycosyl transferases (GTs) were more frequent in *Cyanobacteria* (Kruskal-Wallis test, *p* < 0.05) (Fig. 4a). No statistically significant differences were observed in the frequency of the different CAZyme classes between the different *Flavobacteriaceae* clades (Fig. 4b and Additional file 4). In contrast, the number of degradative CAZymes arranged in PULs per Mbp (Kruskal-Wallis test, *p* < 0.05) and putative PULs per Mbp (Kruskal-Wallis test, *p* < 0.01) revealed a significant variation in the potential to degrade polysaccharides between the *Flavobacteriaceae* clades (Additional file 4). Strains of the Marine clade possessed an average of 42.4 degradative CAZymes per Mbp of which 14.6% were PUL-associated. Moreover, 3.1 PULs per Mbp were identified in the Marine strains with 5.2% being “complete” (Table 3). The rest of the annotated PULs included only polysaccharide binding proteins (*susC/D* genes), but no CAZyme-encoding genes. Within *Flavobacteriaceae*, *Capnocytophaga* genomes encoded 2.5 times more PUL-associated CAZymes (Kruskal-Wallis test, *p* < 0.05) than genomes belonging to the Marine clade. Similarly, strains of the *Capnocytophaga* clade showed on average the highest frequency of PULs (8.4 PULs per Mbp) while Marine, *Tenacibaculum-Polaribacter* and *Flavobacterium* genomes followed with 3.1, 2.9 and 1.8 PULs per Mbp, respectively (Table 3). In the Marine clade, among the most abundant GH families were GH family 74 (GH74) and GH109 (Additional file 4). GH74 comprises many xyloglucan-hydrolysing enzymes, while enzymes belonging to GH109 have α-*N*-acetylgalactosaminidase activity [54]. In the case of CBMs in the Marine clade, most hits belonged to the chitin- or peptidoglycan-binding family (CBM50) and cellulose- and xyloglucan-binding family (CBM44) [54].

**Table 3:** Comparison of frequencies of degradative CAZymes, PULs and CAZymes in PULs in *Flavobacteriaceae* genomes

	*Flavobacteriaceae*			
	M	C	F	T
CAZymes/Mbp	42.4	43.7	28.9	40.1
Total PULs/Mbp	3.1	8.4	1.8	2.9
%Complete PULs/Mbp	5.2	4.7	7.3	5.0
CAZymes in PULs/Mbp	6.2	15.5	4.9	8.5
%CAZymes in PULs/Mbp	14.6	35.1	11.0	19.3

A more detailed table of the PUL-associated CAZymes distribution can be found in Additional file 4

M *Marine*, C *Capnocytophaga*, F *Flavobacterium*, T *Tenacibaculum-Polaribacter*

### Gliding motility and type 9 secretion

**Fig. 5:**
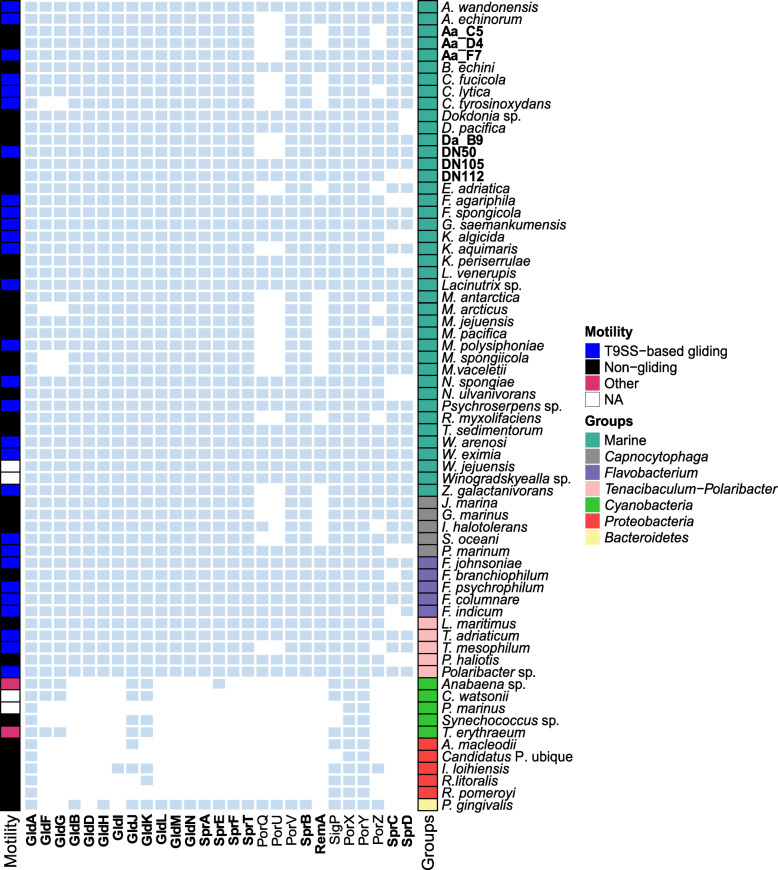
Presence of homologs of gliding motility and type 9 secretion proteins in the predicted proteomes. Presence of homologs is indicated by light blue squares and absence by white squares. Strains sequenced in the framework of this study are indicated in bold. Each species was annotated based on its taxonomy (Groups) and motility as reported in literature. Protein names in bold are considered essential or important for *F. johnsoniae* gliding. The rest of the proteins are either non-essential or their role in *F. johnsoniae* gliding has not been determined [24, 32]. Other, gliding motility mechanism other than T9SS-based; NA, no information available (see also Additional file 3)

Based on the studies of Veith et al. (2017) and McBride (2019), we defined a set of 27 proteins that are related to gliding motility and T9SS, e.g. as reported for *F. johnsoniae* and *P. gingivalis*. These proteins were categorized as i) essential, ii) important, when their absence had a minimal effect but not complete loss of motility and secretion, and iii) non-essential, according to previous reports on *F. johnsoniae* [24, 32] (Additional file 3). Forty-nine publicly available genomes (Additional file 2) of gliding and non-gliding members of the different clades of the *Flavobacteriaceae* (Marine, *Capnocytophaga*, *Flavobacterium*, *Tenacibaculum*-*Polaribacter*) (Fig. 5) isolated from different environments (host-associated and non-host associated) were screened for the presence of homologs to these 27 proteins, using amino acid sequences. All 29 flavobacteria reported in the literature as ‘gliding’ had homologs for 20 of the motility and T9SS-related proteins (GldA, GldB, GldF, GldG, GldD, GldH, GldI, GldJ, GldK, GldL, GldM, GldN, SprA, SprE, SprF, SprT, PorV, SigP, PorX and PorY), except for *Cellulophaga tyrosinoxydans* which was lacking GldF and GldG homologs (Fig. 5). The 20 proteins encoded by the genomes of all other gliding flavobacteria include all the Gld and Spr proteins that were considered essential or important for motility in *F. johnsoniae* (Additional file 3) [22]. Homologs to the motility adhesins RemA were not detected in five out of the 29 genomes of gliding members of the *Bacteroidetes* phylum, while SprB homologs were present in all gliding bacteria. In our dataset, there were two strains for which no information regarding motility was available in literature (*Winogradskyella jejuensis* CP32^T^ and *Winogradskyella* sp. J14–2). Here, we report that both strains have a complete set of homologs to the T9SS-based gliding proteins. For the non-gliding *P. gingivalis*, homologs for the major T9SS components (GldK, GldL, GldM, GldN, SprA, SprE, SprT) were detected in the genome (Fig. 5). Four proteins (GldF, GldG, GldD, GldI) that are required for gliding motility of *F. johnsoniae* did not appear to be encoded in the genome of *P. gingivalis*. The same was the case for the motility adhesin RemA and the SprB supporting proteins, SprC and SprD. No obvious differences in the presence of gliding and T9SS proteins were found among genomes of host-associated and non-host-associated strains (data not shown). Similarly, no specific pattern was observed in terms of the gliding motility of the different *Flavobacteriaceae* clades (Fig. 5).

Apart from the *Bacteroidetes*, proteins encoded by genomes from two other phyla (*Cyanobacteria* and *Proteobacteria*) were searched by BLASTP (E-value 1e-5) for the presence of homologs of gliding and T9SS-related proteins (Fig. 5). In general, homologs to *F. johnsoniae* motility proteins were scarce among these two phyla. From the gliding motility proteins, only GldA homologs were present in all cyanobacterial and proteobacterial genomes. However, GldA is the ATP-binding component of the ABC transporter of the gliding motility apparatus and might share sequence similarity with many other (not necessarily gliding-related) ATP-binding regions of ABC transporters [22]. *Anabaena* sp. and *Trichodesmium erythraeum* strains were the only cyanobacteria in our dataset previously reported as gliding [55, 56], and both genomes were found to encode homologs for five gliding proteins (GldA, GldF, GldG, GldJ, GldK) and three regulatory proteins (SigP, PorX and PorY). The *Anabaena* sp. genome also encoded one Spr ortholog (SprE) that was absent from *T. erythraeum*. Among the genomes tested in this study, those of *Prochlorococcus marinus* and “*Candidatus* Pelagibacter ubique” encoded the lowest number (three) of homologs to the motility and T9SS proteins (Fig. 5).

**Fig. 6:**
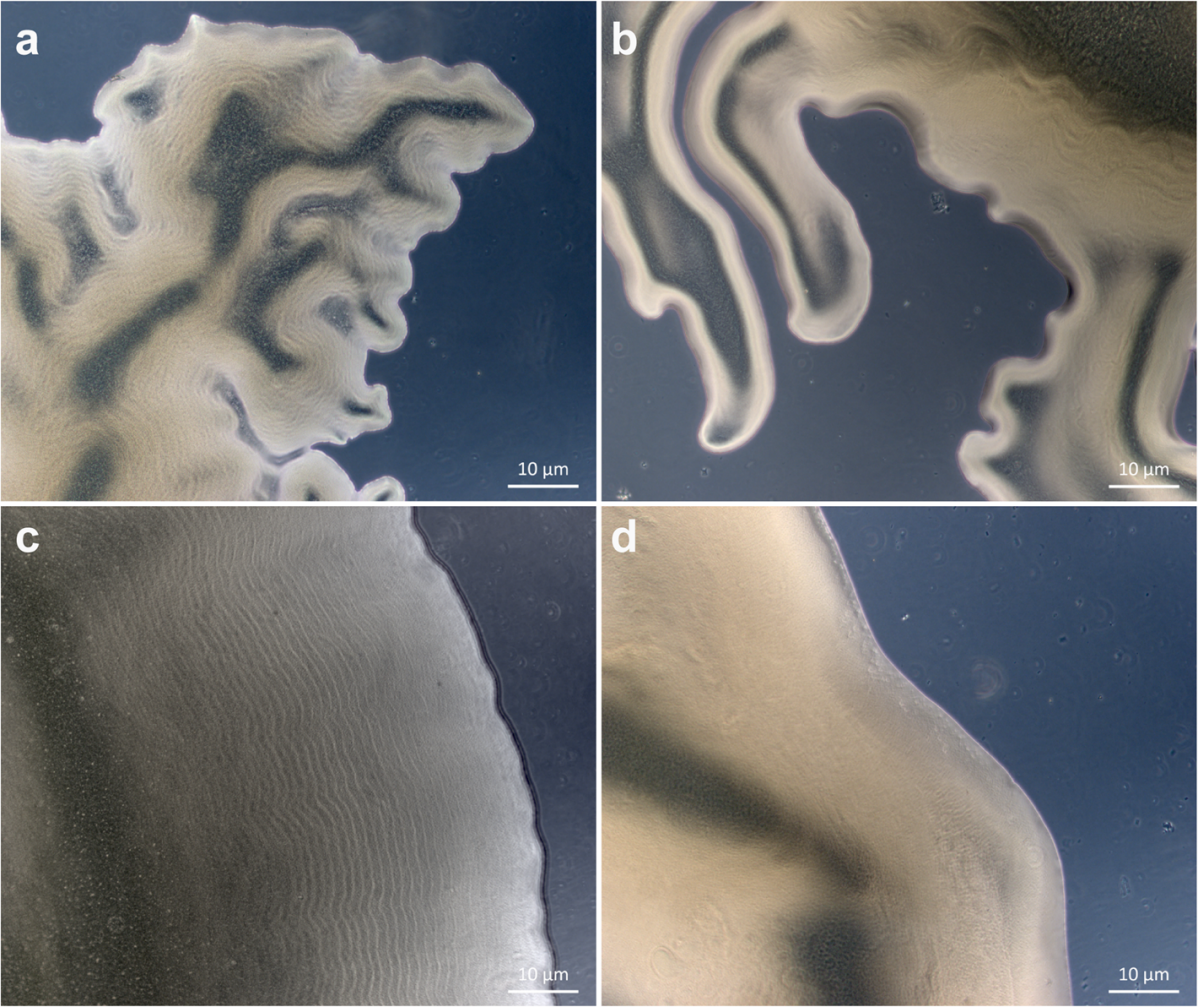
Colony morphologies of the studied flavobacterial strains. **a** and **b** Spreading colonies of strains DN50 and Aa_F7, respectively, that form slender peninsulas on 1% agar; **c** Surface contour pattern of DN50 colonies on 1.8% agar; **d** Non-spreading colonies of DN105. Photos were taken with an AxioCam ICc3 attached to an Axio Scope.A1 (Zeiss) phase-contrast microscope

In addition to examining the genomes of the seven flavobacterial sponge isolates, their gliding motility was also tested based on the formation of spreading colonies on agar. All newly sequenced genomes encode homologs for each of the proteins considered essential for gliding motility and type 9 secretion in *F. johnsoniae* (Additional file 3). However, after examination of the colony morphology, most of the isolated strains (DN105, Da_B9, Aa_C5, Aa_D4, DN112) were non-spreading on agar, and only Aa_F7 and DN50 formed spreading colonies. The edges of the colonies of both Aa_F7 and DN50 formed slender peninsulas spreading on 1% (w/v) agar plates (Fig. 6a, b). On 1.8% (w/v) agar plates, DN50 exhibited a colony surface contour pattern probably attributed to gliding motility and secondary colony formation (Fig. 6c). This pattern was not observed for any of the other strains.

**Fig. 7:**
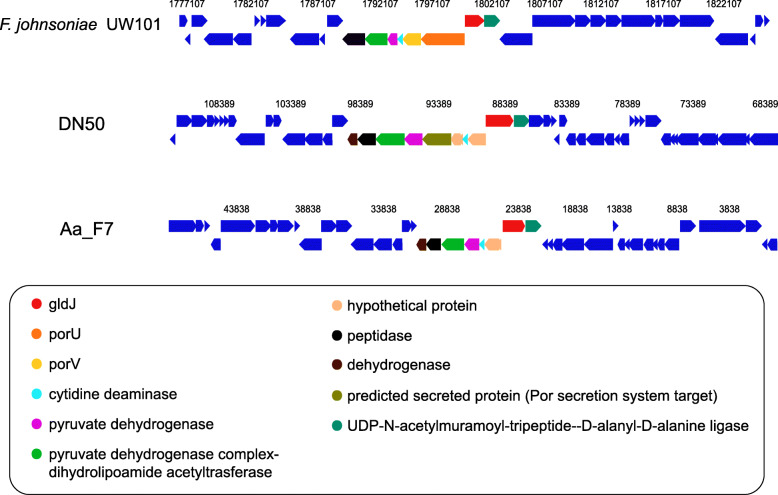
Gene neighbourhoods of *gldJ* in Aa_F7 and DN50 compared to *F. johnsoniae* UW101 genome. Genes of the same colour are from the same orthologous group. Light yellow genes had no Cluster of Orthologous Group (COG) assignment. (Chromosome viewer-IMG/MER)

Homologs for all gliding and T9SS proteins were identified in the genomes of the spreading strains (Aa_F7 and DN50), except for PorQ and PorU. Consequently, the chromosomal neighbourhood of these genes was investigated for the genomes of Aa_F7 and DN50 and compared to that of *F. johnsoniae* UW101 (Fig. 7). In the *F. johnsoniae* genome, *porU* (Fjoh_1556) lies immediately upstream of *porV* (Fjoh_1555) and adjacent to *gldJ* (Fjoh_1557), being encoded on the opposite strand. For Aa_F7 and DN50, the gene arrangement was similar to that found in *F. johnsoniae* with a gene coding for a ligase downstream of *gldJ*. No homolog to *porU* was identified in the genomes of Aa_F7 and DN50, and the genes directly adjacent to *gldJ* were annotated as hypothetical proteins (Fig. 7).

Sequence alignments of these hypothetical proteins and *F. johnsoniae* PorU by BLASTP showed amino acid identities of 24.4 and 26.3% and a query cover of 8 and 14% for Aa_F7 and DN50, respectively. In the case of *porQ*, it was not possible to use the gene neighbourhood because it is not adjacent to any of the known gliding or T9SS-related genes in *F. johnsoniae*. Instead, a BLASTP search with a lower cut-off (E-value 1e-0) revealed weak hits for PorQ in both Aa_F7 and DN50. Interestingly, in the genome of DN105 all components of *F. johnsoniae* gliding and T9SS machinery were identified, even though it did not form spreading colonies on agar under the examined conditions (Fig. 6d). Similarly, *Bizionia echini*, *Lacinutrix venerupis* and *Tamlana sedimentorum* were previously reported as non-motile, but they carry a complete set of orthologous gliding genes in their genomes (Fig. 5).

### Secondary metabolome

The genomic repertoire for secondary metabolite synthesis of the 66 studied bacteria was assessed using the ‘Antibiotics and secondary metabolite analysis shell’ (antiSMASH) [57]. In *Flavobacteriaceae*, the vast majority of BGCs was predicted to encode terpene synthases (44%), followed by type III polyketide synthases (T3PKS) (9%), non-ribosomal peptide synthetases (NRPS) (7%), lanthipeptide (6%) and aryl polyene (6%) clusters. In total, the percentage of genes putatively involved in the biosynthesis of secondary metabolites in relation to the total number of protein-coding genes in each genome ranged between 0.02 and 0.3%. *Cyanobacteria* showed the highest average number of annotated BGCs (*n* = 7), compared to *Flavobacteriaceae* (*n* = 4) and *Proteobacteria* (*n* = 4) (Additional file 1; Table S4). Gene clusters encoding the proteins involved in the biosynthesis of non-ribosomal peptides and polyketides were identified in genomes from all three major groups (Additional file 1; Table S5). Terpene BGCs were detected in all analysed genomes and showed the highest relative abundance in *Flavobacteriaceae* and *Cyanobacteria*. In contrast, the most abundant gene cluster in *Proteobacteria* was affiliated with the biosynthesis of homoserine lactones (HSL), a quorum sensing (QS) molecule in Gram-negative bacteria (Table 4). Interestingly, only one flavobacterial strain (*Muriicola jejuensis* DSM 21206) harboured HSL BGCs.

**Table 4:** Distribution of BGCs (mean number of BGCs per strain) across major taxonomic groups and different *Flavobacteriaceae* clades

Gene cluster type (antiSMASH)	*Flavobacteriaceae*				*Cyanobacteria*	*Proteobacteria*
	M	C	F	T		
Terpene	1.63	1.20	1.40	1.20	1.60	0.40
T3PKS	0.39	0	0.20	0.20	0.20	0
NRPS	0.29	0	0.20	0	1.00	0
Lanthipeptide	0.22	0.40	0	0.20	0	0
Aryl polyene	0.17	0	0.60	0.20	0.60	0.20
Aryl polyene-Resorcinol	0.17	0	0.60	0	0	0
Bacteriocin	0.12	0.20	0.20	0	1.40	0.80
NRPS-like	0.05	0.40	0	0	0.60	0
Siderophore	0	0.20	0.60	0.20	0	0.20
Homoserine lactone	0.02	0	0	0	0	1.40
Betalactone	0	0	0.20	0	0	0.40

Only BGC types with mean abundance of more than 0.1 BGCs/strain in the complete dataset are shown. A more detailed table of the BGC distribution can be found in Additional file 1; Table S5

M *Marine*, C *Capnocytophaga*, F *Flavobacterium*, T *Tenacibaculum*-*Polaribacter*

Within the *Flavobacteriaceae*, strains from the Marine and *Flavobacterium* clades contained the largest average number of BGCs (Additional File 1; Table S4), whereas on average only 2 BGCs were detected in the *Tenacibaculum*-*Polaribacter* genomes. In general, the different flavobacterial clades exhibited a similar composition in terms of the types of predicted BGCs being enriched in their genomes, including terpene, lanthipeptide, NRPS and aryl polyene BGCs.

**Fig. 8:**
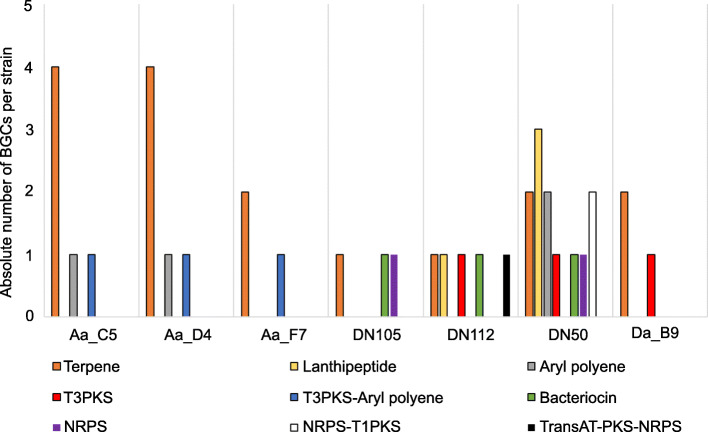
Composition of BGCs identified in the genomes newly sequenced in this study. The absolute number of BGCs per strain assigned to each BGC class is shown. Different colours indicate different BGC classes

Genome mining for BGCs of the seven newly isolated flavobacteria resulted in the detection of five BGCs, on average, belonging to six different classes based on the antiSMASH database. In total, 63% of the identified BGC encoded proteins were predicted to be involved in pathways for the production of terpenes, lanthipeptides and aryl polyenes. Less abundant but widely distributed across these genomes were bacteriocin, T3PKS and NRPS BGCs. Strain DN50 harboured the largest number of different BGC types and the highest absolute BGC abundance (Fig. 8). A more detailed comparative analysis of the BGCs showed that the necessary proteins predicted to synthesize carotenoid pigments were present in all seven flavobacterial genomes. Similarly, DN50 harboured a BGC related to the production of flexirubin, another type of bacterial pigment [1, 2, 58]. To accompany the genome mining results, antimicrobial activity of the seven sponge-associated flavobacterial strains was tested against six indicator strains (*Escherichia coli*, *Bacillus subtilis*, *Staphylococcus simulans*, *Aeromonas salmonicida*, *Candida oleophila*, *Saprolegnia parasitica*) belonging to the three categories Gram-positive, Gram-negative bacteria and fungi-oomycetes. Even though the genome predictions revealed a variety of secondary metabolite BGCs, none of the studied isolates showed antimicrobial activity under the examined conditions (data not shown).

## Discussion

The family *Flavobacteriaceae* in the phylum *Bacteroidetes* currently contains more than 150 described genera [6] that are widespread in marine and non-marine ecosystems [8]. Thriving in such diverse habitats could indicate a high genetic plasticity. Disentangling the phylogeny of the family was controversial and challenging in the past, yet it has remained relatively stable for the past two decades [1]. In this study, we followed the definition of flavobacterial clades as proposed by McBride (2014) with a focus on the Marine clade. Whole-genome reconstructed phylogeny based on 120 single copy bacterial marker genes confirmed the 16S rRNA gene-based phylogeny by placing all newly sequenced strains in the Marine clade of the family *Flavobacteriaceae*.

We compared representative genomes of phylogenetically diverse flavobacteria inhabiting various niches to reveal novel insights into the characteristic properties of this group. The functional profiling based on Pfam entries revealed a high degree of distinction between the *Flavobacteriaceae* in comparison with other taxa dominant in marine environments, i.e. *Proteobacteria* and *Cyanobacteria*. This was also true for the genomic content of flavobacteria from different clades. However, comparison of functional profiles of host-associated and non-host-associated flavobacteria did not show significant dissimilarity. These results indicate that the functional traits within the *Flavobacteriaceae* were congruent with both single-copy marker gene- and 16S rRNA gene-based phylogeny [59, 60], rather than the habitat type, i.e. host-associated or non-host-associated [60]. However, it is important to mention that the environmental or organismal source of bacterial strains that have been isolated under laboratory conditions does not prove host-attachment in situ. Moreover, in marine environments a considerable number of host-associations might be coincidental as many microbes show metabolic versatility of a biphasic lifestyle-strategy that includes seawater particles (“free-living”) and animal hosts [61–64], most likely in the search of nutrient sources [64]. Previous studies provided evidence with respect to the phylogenetic conservation of traits associated with organic substrate utilization in different microorganisms [65, 66] and particularly in members of the *Flavobacteriaceae* [59, 60]. Interestingly, functional traits appeared to be highly conserved within the different flavobacterial clades (48% shared Pfams), even though the isolation sources of the strains were largely diverse. The key functions strongly enriched in *Flavobacteriaceae*, in general, and also in the Marine clade, were related to nutrient acquisition in regard to complex carbohydrate metabolism as well as gliding motility.

These two features have been repeatedly linked to the important role of *Flavobacteriaceae* in the degradation of high molecular weight organic matter within the marine environment [8, 16]. Investigation of the repertoire of polymer-degrading enzymes among dominant marine bacterial groups revealed that *Flavobacteriaceae* had significantly more GHs, PLs, CEs and CBMs per Mbp compared to *Cyanobacteria* and *Proteobacteria*, supporting their pronounced capacity as polymer degraders and perhaps as key players in ocean carbon cycling [9, 16, 20, 59, 60]. The capacity is justified by *Bacteroidetes*’ particular genomic content which is enriched in genes encoding highly specific CAZymes, regulatory proteins and transporters arranged in clusters termed PULs that are required for depolymerization of complex polysaccharides [67, 68]. Previous analyses of the PUL spectrum in marine *Flavobacteriaceae* revealed a similar average number of degradative CAZymes and PULs [20]. Between the different flavobacterial clades, *Capnocytophaga* strains possessed more PULs and PUL-associated CAZymes per Mbp, likely reflecting their increased metabolic capabilities. This was also supported by their slightly larger genomes compared to the Marine clade strains. Genome size could be partially associated with the different ecological niches colonized by *Capnocytophaga* and Marine flavobacteria, as bacteria living in habitats with ample nutrient supply tend to have larger genomes and target more complex substrates [69].

Regarding the function of the predicted CAZymes in the Marine clade genomes, the GH74 family occurs with the highest frequency and contains various enzymes that hydrolyse *β*-1,4 linkages of glucans. Thus, these enzymes assist in the degradation of different oligo- and polysaccharides, including xyloglucans [54],which is a hemicellulose polysaccharide present in plant cell walls and green algae [70]. GH109, predicted as *α*-*N*-acetylgalactosaminidase, was the second most frequent GH family in the Marine clade genomes. These enzymes cleave *N*-acetylgalactosamine residues from various substrates such as glycolipids, glycopeptides and glycoproteins. It is highly likely that these substrates are derived from dissolved and/or particulate organic matter found in seawater, but also from the matrix of sponges [71, 72]. In addition, high frequencies of CBM50 and CBM44 genes were observed in the Marine clade. CBM50 proteins are found attached to enzymes that might cleave either bacterial peptidoglycan or animal chitin, and CBM44 are modules related to enzymes that bind both cellulose and xyloglucan [9, 72]. Taken together, our results reflect that the Marine clade flavobacteria have the genetic repertoire for utilizing a large diversity of carbon sources derived from algae, plants, bacteria and animals. This feature common in these microbes derived from numerous source habitats underlines the essential role of substrate utilization in the colonization of both host-associated and non-host-associated niches.

Many members of the *Bacteroidetes* and particularly *Flavobacteriaceae* can glide rapidly over surfaces using two novel and intertwined machines, one gliding motor involved in cell movement and one protein secretion system, known as T9SS [22, 24, 26, 33]. Gliding is common among members of the *Flavobacteriaceae* [73]. This notion could be reinforced by the fact that genomes of all investigated gliding flavobacteria encoded homologs of the necessary components for T9SS-based gliding, with the exception of *Cellulophaga tyrosinoxydans*. This bacterium was lacking the two proteins GldF and GldG, which together with GldA are thought to be the components of an ATP-binding cassette (ABC) transporter involved in flavobacterial gliding [74]. According to McBride and Zhu (2013), two other flavobacteria, *Cellulophaga algicola* and *Maribacter* sp. HTCC2170 were also actively gliding even though their genomes were lacking the genes encoding this ABC transporter [22]. This might suggest either the involvement of another ABC transporter, common in most organisms, or a non-essential role of this ABC transporter in *Bacteroidetes* gliding motility [22, 24, 75]. The T9SS exports the motility adhesins RemA and SprB which are propelled along the cell surface by the gliding motor, resulting in cell movement of *F. johnsoniae* [22, 26, 76]. SprB homologs were ubiquitous in gliding flavobacteria, whereas RemA was rare. This might indicate that SprB is more important for *Bacteroidetes* gliding. Additional novel motility adhesins may allow other species to move over diverse surfaces [26, 75].

Gliding motility is considered as an efficient strategy to enhance survival, but the actual role in nature is still unknown. It has been previously suggested that gliding over surfaces facilitates access to nutrient sources [23], or is involved in pathogenesis [30] or symbiosis [77]. Accordingly, in both non-host- and host-associated bacteria, the importance of T9SS has been demonstrated for many processes such as polymer degradation, adhesion, motility and virulence [24]. In this study, no specific link was found between host-association and gliding or type 9 secretion. Similarly, no difference was found when comparing the genomic repertoire of members of different taxonomic clades within the *Flavobacteriaceae*. These observations add to the fact that flavobacteria appear to be ‘generalists’, rather than ‘specialists’ and this might explain why they thrive in such diverse niches. Especially in the marine environment, the availability of nutrients might drive opportunistic host-microbe interactions, which might explain the presence of flavobacteria in the biomes of numerous marine hosts.

The high degree of conservation of Gld and Spr proteins in all *Flavobacteriaceae*, irrespective of their environmental source or taxonomic clade, supports the notion that T9SS-based gliding motility is widespread among *Bacteroidetes* members [22] and especially among *Flavobacteriaceae*. Gliding motility occurs in other bacteria apart from the phylum *Bacteroidetes* (myxobacteria, cyanobacteria, mycoplasmas, etc.) but these employ their own unique machineries substratum-fixed protein complexes (A-motility), type IV pili (T4P) or membrane protrusions [78–81]. Filamentous cyanobacteria such as *Anabaena* sp. and *Trichodesmium erythraeum* are thought to share a T4P-like nanomotor and polysaccharide secretion that drive and facilitate locomotion [78, 79]. Nevertheless, homologs to certain core *Bacteroidetes* gliding proteins (GldA, GldF, GldG, GldI, GldJ, GldK, SprE) were found to be encoded in genomes belonging to *Cyanobacteria* or *Proteobacteria*, regardless of their gliding status or mechanism. For example, GldA, GldF and GldG are similar to many putative components of the ABC transporter system, which is ubiquitous in all microorganisms [22, 33]. These proteins might serve another purpose in the respective organisms that might not be linked to gliding motility. In general, the paucity of homologs to *Bacteroidetes* gliding and T9SS proteins among bacteria from other phyla implies the presence of diverse and likely unrelated gliding motility mechanisms [22, 24, 82].

Gliding motility can also result in colony spreading, observed on agar plates as flat, irregularly edged colonies [1, 22, 26]. The majority of the strains investigated for colony spreading (5 out of 7) were non-spreading on agar under the tested conditions, even though presence of all genes encoding proteins involved in the gliding motility apparatus was predicted by the genomic analysis. Presence of a gene in a genome does not yet imply gene expression. As described above, several bacterial strains that were previously described as ‘non-gliding’ had all essential genes present in their genomes.

Moreover, previous findings support that formation of non-spreading colonies is not necessarily an indication of absence of gliding motility. For example, *Maribacter sp.* HTCC2170 could glide well on glass but its colonies did not spread on agar [22]. In addition, it should be highlighted that the growth media used in our colony spreading tests were high in nutrients. Spreading under low nutrient conditions is more effective because non-metabolizable sugars tend to supress the active cell movement on agar media [1, 83, 84]. Thus, these bacteria may be motile in other conditions that were not examined here. On the other hand, the strains Aa_F7 and DN50, both carrying homologs for all T9SS-gliding proteins in their genomes, formed spreading colonies on agar displaying characteristic edges and a surface pattern indicative of secondary colony formation (Fig. 6). In contrast to the other strains, it is possible that these isolates could metabolize all sugars present in the high-nutrient media, thus surpassing the carbohydrate-mediated inhibitory effect on colony spreading. The absence of homologs to PorQ and PorU from Aa_F7 and DN50 genomes did not affect their gliding motility. Both PorQ and PorU are involved in the T9SS substrate attachment to the cell surface in *P. gingivalis* [85, 86] but in *F. johnsoniae* their deletion did not affect gliding motility [87]. This might be another indication that PorQ and PorU are not essential for full motility [24, 32].

A wide variety of bioactive molecules, such as antibiotics, cell growth-promoting, antioxidative and neuroprotective compounds, have been previously isolated from members of the *Flavobacteriaceae* [1]. Nevertheless, the metabolic potential of flavobacteria in terms of bioactivity has been underreported in literature. Across taxonomic groups, terpene BGCs were prevalent in the predicted metabolite arsenal of both *Cyanobacteria* and *Flavobacteriaceae*, particularly of the Marine clade. Terpenes are the largest class of small-molecule natural products, found in almost all life forms and performing diverse functions [88]. Previous findings showed that terpene synthases are widely distributed in bacteria, the majority of which are Gram-positive (*Actinomycetales*) but also in Gram-negative bacteria, such as *Cyanobacteria* and *Flavobacteriales* [89]. In terms of bioactivity, to date there are only a few reports on terpenes of microbial origin exhibiting antimicrobial [90–92] and antioxidant activity [45]. The almost complete absence of HSL BGCs from the *Flavobacteriaceae* genomes, compared to their high relative abundance in *Proteobacteria* (Table 4), is in accordance with previous studies on flavobacteria that suggest the existence of alternative QS signalling molecules for *Bacteroidetes* [93, 94]. For example, strains of *Zobellia galactanivorans* (member of *Flavobacteriaceae*) were found to synthesize another type of QS signalling molecules belonging to dialkylresorcinols, a class of natural products with antibiotic and antioxidant activity [94]. In addition, the detection of a HSL-degrading enzyme in the genome of *Z. galactanivorans* [15] implies the presence of a communication system distinct from the HSL-based QS system that could function as a defence mechanism with antibiotic effect [94]. Within *Flavobacteriaceae*, the secondary metabolite repertoire did not appear to be influenced by phylogeny, as the global composition of predicted BGCs was similar among the clades. Besides terpene synthases, BGCs responsible for the biosynthesis of lanthipeptides were also found in high abundance in flavobacterial genomes. Formerly known as lantibiotics, they are ribosomally synthesized post-translationally modified peptides that belong to class I bacteriocins [95]. It has been previously suggested that bacteriocins play a critical role in mediating microbial community dynamics [96, 97]. Presumably, bacteriocin-producing flavobacteria act as anti-competitors, facilitating the invasion of other closely related species into an established microbial community to successfully colonize a niche. An additional role suggested for bacteriocins is their involvement in host defence, protecting the host from pathogens [97].

Genome mining of the BGC arsenal of the sponge-associated strains sequenced in this study revealed homologs to carotenoid-producing BGCs in all seven genomes. Carotenoids are often the most abundant pigments in marine flavobacteria, and their importance lies in their strong antioxidant properties. In flavobacteria, a few structurally rare carotenoids have been identified before, such as saproxanthin and myxol that show significant antioxidative activities and neuroprotective effects [45]. Flexirubins represent another type of pigment common in *Bacteroidetes* but not in other bacteria [1]. A BGC similar to one shown to encode proteins needed for flexirubin production was identified in strain DN50. Interestingly, such pigments exhibit anticancer and antimicrobial properties (e.g. against *Mycobacterium* sp.) and are considered promising candidates for treatment and prevention of cancer and microbial infections [98, 99].

Even though the genome predictions revealed a large number and variety of secondary metabolite BGCs, none of the studied isolates showed antimicrobial activity under the conditions we examined. The majority of these biosynthetic loci are frequently dormant or expressed at low constitutive levels under laboratory conditions, keeping the true biosynthetic potential of microorganisms hidden and thus hampering the discovery of novel bioactive compounds [100, 101]. This ‘silent’ state can be reversed by inducers of gene expression, such as environmental cues, nutrients or signal molecules [100, 101]. Unlocking the full metabolic potential encoded by the studied strains might require high-throughput screening of various growth conditions in combination with a large number of indicator strains.

## Conclusions

The comparative genomics analysis performed in this study demonstrated that 16S rRNA gene- and single-copy marker gene-based phylogeny, rather than life strategy of the organisms is the main factor correlated to the functional profile of *Flavobacteriaceae*. The traits responsible for the functional divergence between phyla investigated here were found to be associated with gliding motility and nutrient acquisition through the catabolism of carbohydrates. Marine flavobacteria appear to be potent utilizers of a large variety of carbon polymers from algae, bacteria, plants and animals, confirming their role in the ocean carbon cycling as exceptional degraders of particulate organic matter. Additionally, inspection of the gene content revealed the occurrence of homologs for all major components of the T9SS-gliding motility apparatus in all *Flavobacteriaceae* in contrast to members of other phyla (*Cyanobacteria* and *Proteobacteria*) that are known to use different mechanisms for gliding. Phenotypic assays showed the formation of spreading colonies for some of the tested flavobacteria that had the complete set of T9SS-gliding homologs, confirming that not all potentially gliding bacteria form spreading colonies on agar. In terms of their secondary metabolic potential, a large diversity of BGCs was identified in the studied genomes, with terpene BGCs being highly prevalent in both *Flavobacteriaceae* and *Cyanobacteria*. Other BGCs that potentially encode proteins required for the production of compounds with known antimicrobial, antioxidant and anticancer properties were found in *Flavobacteriaceae*. Nevertheless, bioactivity tests did not reflect these genomic findings supporting the fact that the true biosynthetic potential of microorganisms remains hidden due to the ‘dormant’ state of gene clusters under laboratory conditions. Hence, while this study provides the required broad overview of the genomic content of *Flavobacteriaceae* in terms of their carbon metabolism, gliding motility and secondary metabolite biosynthetic potential, further studies are essential to enhance our current understanding of these distinct features and how flavobacteria implement them in their natural environment.

## Methods

### Sample collection and isolation of strains

Samples from the sponges *Aplysina aerophoba* and *Dysidea avara* were collected in January 2012 and June 2014, respectively, from Cala Montgó, Spain (42° 06′ 52.20″ N, 03° 10′ 06.52″ E) by SCUBA diving at a depth of approximately 12 m [50, 51]. The collection of sponge samples was conducted in strict accordance with Spanish and European regulations within the rules of the Spanish National Research Council with the approval of the Directorate of Research of the Spanish Government. The study was found exempt from ethics approval by the ethics commission of the University of Barcelona since, according to article 3.1 of the European Union directive (2010/63/UE) from the 22/9/2010, no approval is needed for sponge sacrifice, as they are the most primitive animals and lack a nervous system. Moreover, the collected sponges are not listed in the Convention on International Trade in Endangered Species of Wild Fauna and Flora (CITES). Tissue preparation and cryopreservation were performed as previously described [102]. Cryopreserved samples were stored at − 80 °C. Initial cultivation and isolation of the *Flavobacteriaceae* strains from the sponges *Aplysina aerophoba* (DN50, DN105, DN112, Aa_C5, Aa_D4 and Aa_F7) and *Dysidea avara* (Da_B9) were described in Versluis et al. (2017) and Gutleben et al. (2020) [50, 51] (Additional file 1; Table S1).

### DNA extraction, identification and sequencing

Glycerol stocks of the original strains were initially used as inoculum for regrowth on the original solid isolation media at 20 °C (Additional file 1; Table S1). Single colonies were picked and cultured in marine broth 2216 (Difco, Detroit, USA) at 30 °C. Genomic DNA was extracted using the MasterPure DNA Purification Kit (Epicentre, Madison, USA). The quality, purity and concentration of the extracted DNA were estimated by gel electrophoresis, spectrophotometric analysis using a NanoDrop 2000c spectrophotometer (Thermo Fisher Scientific, USA) and Qubit dsDNA BR Assay kit (Molecular Probes, Life Technologies) used with the DS-11 FX Fluorometer (DeNovix, USA).

To confirm the identity of the strains, 16S ribosomal RNA (rRNA) gene amplicons were generated by PCR using primers 27F (5′-AGAGTTTGGATCMTGGCTCAG-3′) and 1492R (5′-CGGTTACCTTGTTACGACTT-3′). The PCR reaction mixture contained 10 μL 5X GoTaq reaction buffer (Promega), 1 μL dNTPs (10 mM), 2.5 μL primer 27F (10 μM), 2.5 μL primer 1492R (10 μM), 0.5 μL GoTaq Polymerase (5 U/μL) (Promega) and 1 μL of the extracted DNA. Nuclease-free water (Promega) was added to reach a total reaction volume of 50 μL. The following conditions were used for the bacterial 16S rRNA gene amplification: initial denaturation at 98 °C for 10 min followed by 35 cycles of denaturation at 98 °C for 20 s, annealing at 52 °C for 20 s, elongation at 72 °C for 45 s and a final extension step at 72 °C for 5 min. PCR products were purified using the GeneJET PCR purification kit (Thermo Fisher Scientific, USA) and quantified using a Nanodrop 2000c spectrophotometer (Thermo Fisher Scientific, USA). The purified PCR products were sent for Sanger sequencing with primers 27F and 1492R (GATC Biotech, Cologne, Germany; now part of Eurofins Genomics Germany GmbH). Trimming (99% good bases, quality value > 20, 25-base window) and contig assembly were conducted with DNA Baser (version 3.5.4.2).

Genome sequencing of strains DN50, DN105 and DN112 was performed using the Illumina MiSeq platform (paired end, 2 × 300 bp reads) [50]. The genomes of strains Da_B9, Aa_C5, Aa_D4 and Aa_F7 were sequenced with Illumina HiSeq (paired end, 2 × 150 bp reads). All genomes were sequenced at GATC Biotech (Konstanz, Germany; now part of Eurofins Genomics Germany GmbH).

### Genome assembly and quality control

The quality of the reads was assessed with FASTQC 0.11.4 [103]. Trimmomatic 0.32 was used to remove the Illumina TruSeq adapter sequences and to perform quality filtering [104]. A sliding window trimming approach was employed where part of the read in the window (4 bases) was cropped if the average Phred quality in the window was lower than 20. Any raw reads shorter than 20 bases were discarded. Genome sequences generated by the Illumina MiSeq platform were de novo assembled with the A5-miseq assembler (version 20160825) [105] using default settings. In the case of DN50, the A5-miseq assembler generated a highly fragmented assembly, and the SPAdes 3.11.1 assembler [106] was used instead. For the HiSeq data, the best k-mer size was automatically selected by KmerGenie 1.6741 [107]. SPAdes 3.11.1 was employed for the de novo assembly of the Illumina HiSeq reads using the selected k-mer. BLASTN [108] with default settings was employed to investigate the assemblies for contamination. All contigs assigned to sequencing artifacts and contamination (e.g. *Enterobacteria* phage phiX174) were discarded prior to downstream analysis. Bowtie2 2.2.5 [109] was used to map the quality-filtered reads to the assembled contigs resulting in a sequence alignment map (SAM) file. The SAM file was converted into a binary alignment map (BAM) file that was sorted and indexed using SAMtools 0.1.19 [110]. The BAM file was used as input to improve the draft assemblies using Pilon 1.13 [111]. In addition, coverage per base was calculated using the ‘genomecov’ command of BedTools 2.17.0 [112]. The quality of the draft assemblies was evaluated using QUAST 4.6.3 [113]. Completeness and contamination of all analysed genomes were estimated using CheckM 1.07 with the default set of marker genes [114].

### Genome annotation and comparative genomic analysis

The draft assemblies of the seven strains sequenced in this study were uploaded to the Integrated Microbial Genomes and Microbiomes (IMG/M version 5.0) system [115], and metadata was submitted to the Genomes OnLine Database (GOLD) [116]. For the comparative analysis, 59 genomes of strains belonging to the phyla *Bacteroidetes* (family *Flavobacteriaceae*), *Cyanobacteria* and *Proteobacteria* (Additional file 2) were selected that were publicly available at IMG/M [115]. Protein functional families were automatically assigned via the IMG/M pipeline by comparing predicted proteins to Pfam-A [117] Hidden Markov Models using HMMER 3.0b2 [118]. Gliding motility and T9SS proteins of the gliding *F. johnsoniae* and non-gliding *P. gingivalis* (Additional file 3) were used to identify homologs in the protein sequences of the studied strains by BLASTP searches with an E-value cut-off of 1e-5 using the IMG BLAST Tool. All selected genomes were downloaded via the IMG/M website for further annotation. Predictions of the protein sequences were obtained using Prokka 1.13 [119]. CAZymes were annotated based on HMMER searches (HMMER 3.0b) [118] against the dbCAN database release 6.0 [120]. Annotation of PULs was performed using the PULPy pipeline [121]. PULs which contained one *susC/susD* gene pair and at least one adjacent gene coding for CAZymes were assigned as “complete”. The online server antiSMASH 5.0 [57] was used for the identification of secondary metabolite BGCs with “relaxed” detection strictness. The ClusterBlast and KnownClusterBlast modules integrated into antiSMASH 5.0 [57] were also used for comparative gene cluster analysis based on the NCBI GenBank [122] and the ‘Minimum Information about a Biosynthetic Gene Cluster’ (MIBiG) [123] data standard, respectively.

### Phylogenetic analysis and data selection

Taxonomic assignment of the seven newly sequenced isolates was performed by: 1) BLASTN (May 2019) [108] of near full length 16S rRNA gene sequences recovered from this study against the nr/nt NCBI database and 2) single-copy marker gene analysis and placement of genomes in the Genome Taxonomy Database (GTDB) reference tree [124] using the GTDB-Tool Kit 1.1.0 (GTDB-Tk) *classify* workflow [125]. For the reconstruction of the phylogeny, the closest relative of each isolate in NCBI was selected based on the availability of genomes in IMG/M [115]. Similarly, representatives of the other clades of the family *Flavobacteriaceae* were chosen according to the genome availability in IMG/M [115], but also the phylogenetic position of the isolates (based on their 16S rRNA gene sequences) in ARB [126] using the SILVA SSU Ref NR 99 database (release 132) [127] as reference. To compare the functional traits of the *Flavobacteriaceae* members, representatives of two other phyla that are dominant in the marine environment (*Cyanobacteria* and *Proteobacteria*) were included in the analysis and were also used as outgroup. Multiple alignments of the 16S rRNA gene sequences were performed using the SINA Aligner 1.2.11 [128]. A maximum likelihood tree was generated in ARB [129] employing RAxML 7.0.3 [130] with 1000 iterations of rapid bootstrapping. Phylogenomic analysis was performed using GTDB-Tk 1.1.0 [125]. A concatenated amino acid-based phylogeny was reconstructed using the translated amino acid sequences of 120 bacterial marker genes identified in the studied genomes and aligned by GTDB-Tk *identify* and *align* module, respectively. The resulting multiple sequence alignment was used for generating a maximum likelihood protein tree using FastTree 2.1.11 with default parameters [131]. Visualization of both maximum likelihood trees was performed using the Interactive Tree of Life (iTOL) version 3 [132].

### Phenotypic assays

#### Gliding motility tests

To determine colony spreading, two different types of marine agar plates were prepared using marine broth 2216 (Difco, Detroit, USA) solidified with either 1% or 1.8% of Noble Agar (Sigma-Aldrich). The cells were first grown in marine broth at 30 °C until they reached mid-exponential phase. Subsequently, a 5 μL sample of the cell suspension was spotted at the centre of each test plate using an Eppendorf pipette. After observing growth, the edges of the colonies were viewed with an Axio Scope.A1 (Zeiss) phase-contrast microscope at a magnification of 10X. Pictures of the colony edges were taken using an AxioCam ICc3 (Zeiss) attached to the phase-contrast microscope.

#### Antimicrobial activity tests

Disc diffusion assays were carried out according to the Kirby-Bauer susceptibility method [133]. To determine the antimicrobial activity of the seven isolates (Additional file 1; Table S1), a number of indicator strains were used (Additional file 1; Table S6). Prior to the screening, broth cultures of the indicator strains were prepared using 200 μL of the stock cultures to inoculate 3 mL of the respective media and incubated overnight at the corresponding temperatures (Additional file 1; Table S6). Subsequently, 200 μL of the cultures (adjusted to OD_600_ 0.5) were uniformly spread on agar plates using a sterile L-shape spreader. The tested isolates were cultured in marine broth 2216 (Difco, Detroit, USA) at 30 °C until they reached stationary phase. After harvesting, the cultures were centrifuged at 1.657 x g for 20 min at room temperature. The supernatant was sterile-filtered using a 0.2 μm syringe filter to remove the bacterial cells. For screening, sterile, 6-mm diameter filter paper discs were impregnated with 60 μL of the cell-free supernatant and air-dried for 1 h. The paper discs were then transferred onto the agar plates covered with a lawn of the indicator strains. As positive controls, antibiotics known to be effective against the indicator strains were used (Additional file 1; Table S6). The uninoculated growth medium of each of the tested strains was used as negative control, after receiving the same treatment as described for the cultures of the isolates. The plates were incubated for 24 to 48 h at different temperatures depending on the indicator strains used for the assay. After the incubation, the plates were examined for the presence of inhibition zones that were measured using a calliper. All assays were performed in triplicate.

### Statistical analysis

Data were analysed and visualized in R 3.5.0 using vegan 2.5–2 [134], phyloseq 1.26.1 [135], ggplot2 3.1.1 [136], VennDiagram 1.6.20 [137], and ComplexHeatmap [138]. Functional comparisons between genomes were performed using Pfam annotations as input data. A Bray-Curtis dissimilarity distance matrix was calculated based on the relative abundance (Gene counts/Total genes) of Pfam profiles with the ‘vegdist’ function in the vegan R package. Variation in the functional profiles was assessed by non-metric multidimensional scaling (NMDS) ordination using the Bray-Curtis dissimilarity matrix with the ‘ordinate’ function in the phyloseq R package. NMDS plots were created using the ‘plot_ordination’ function of the ggplot2 R package. The significance of the differences in functional profiles across major taxonomic groups and within the *Flavobacteriaceae* was tested by non-parametric permutational analysis of variance (PERMANOVA) on the Bray-Curtis dissimilarity matrix using the ‘adonis’ function in the vegan R package, with the number of permutations set at 999. To rank the Pfams contributing the most to the differentiation of the functional profiles between genomes, Similarity Percentage (SIMPER) analysis was employed with the ‘simper’ function of the vegan R package. For the pairwise comparisons, only Pfam entries with the highest significant contribution to the dissimilarity are shown (> 0.2%, *p* < 0.05).

## Supplementary information

**Additional file 1: Figure S1.** Maximum likelihood tree of the 66 genomes based on single-copy marker proteins. Phylogeny was inferred from the concatenation of 120 conserved amino-acid sequences by GTDB-Tk. Black circles in the middle of the branches represent Shimodaira-Hasegawa (SH) likelihood support values. Colour annotations represent the different clades and phyla. Sequences belonging to *Cyanobacteria* and *Proteobacteria* were used as outgroups. Names in bold indicate sequences generated in the present study. Scale bar represents amino acid substitutions per site. **Table S1.** Strains and growth media. Details on the preparation of the media and the cultivation conditions can be found in the References section. **Table S2.** Taxonomic assignment of flavobacterial strains sequenced in this study. Information on the 16S rRNA gene sequence of each strain, BLASTN best hits against nr/nt NCBI database and GTDB-Tk classification. **Table S3.** Pfam entries most strongly contributing to differentiating genomes from different *Flavobacteriaceae* clades. Pfam entries with the highest significant contribution (> 0.2%, *p* < 0.05) to the dissimilarity are shown. M, Marine; C, *Capnocytophaga*; T, *Tenacibaculum-Polaribacter*; F, *Flavobacterium*. **Table S4.** Number of BGCs, average gene counts and % of genes in BGCs per group. **Table S5.** Distribution of identified BGCs across different groups and *Flavobacteriaceae* clades. M, Marine; C, *Capnocytophaga*; F, *Flavobacterium*; T, *Tenacibaculum*-*Polaribacter*. **Table S6.** Cultivation conditions of indicator strains and antibiotics used in the antimicrobial activity tests.

**Additional file 2:.** Publicly available genomes used in this study.

**Additional file 3: **List of proteins involved in gliding motility and type 9 secretion in *F. johnsoniae* UW101 and *P. gingivalis* W83 used in the comparative genomics analysis.

**Additional file 4: **Frequencies and Abundances of CAZymes and PULs in different taxonomic groups and *Flavobacteriaceae* clades.

## Data Availability

The raw sequencing data of the genomes [139–145] and the partial 16S rRNA gene sequences [146] of the strains isolated in this study were deposited in the European Nucleotide Archive (ENA) under accession number PRJEB35092 [147]. The draft assemblies and metadata of the strains sequenced in this study are publicly available at the Joint Genome Institute (JGI) Genome Portal under IMG Taxon IDs 2806311042 (Aa_C5), 2806311043 (Aa_D4), 2806311049 (Aa_F7), 2806311064 (Da_B9), 2808606303 (DN50), 2808606304 (DN105), 2808606305 (DN112) and GOLD Study ID Gs0135980. Additional information on all assemblies used in the comparative genomics analysis are included in Additional file 2. All codes and script used for the analyses are available at GitHub (https://github.com/mibwurrepo/Gavriilidou_et_al_Flavos_ComparativeGenomics).

## Abbreviations

PULs: Polysaccharide Utilization Loci
rRNA: Ribosomal RNA
GHs: Glycoside Hydrolases
CAZymes: Carbohydrate-Active enZymes
Sus: Starch utilization system
PLs: Polysaccharide Lyases
CEs: Carbohydrate Esterases
CBMs: Carbohydrate Binding Modules
T9SS: Type 9 Secretion System
BGCs: Biosynthetic Gene Clusters
CDS: CoDing Sequences
Pfams: Protein families
AAI: Amino Acid Identity
GTDB: Genome Taxonomy DataBase
CTD: C-Terminal Domain
GTs: Glycosyl Transferases
antiSMASH: Antibiotics and Secondary Metabolite Analysis SHell
T3PKS: Type III Polyketide Synthases
NRPS: Non-Ribosomal Peptide Synthetases
HSL: HomoSerine Lactones
QS: Quorum Sensing
ABC: ATP-binding cassette
T4P: Type IV Pili
CITES: Convention on International Trade in Endangered Species of Wild Fauna and Flora
IMG/M: Integrated Microbial Genomes and Microbiomes
GOLD: Genomes OnLine Database
MIBiG: Minimum Information about a Biosynthetic Gene Cluster
GTDB-Tk: Genome Taxonomy DataBase-Tool Kit
iTOL: Interactive Tree Of Life
