# Supplementary material for: Comparative genomic analysis of Flavobacteriaceae: insights into carbohydrate metabolism, gliding motility and secondary metabolite biosynthesis
Source: BMC Genomics. 2020 Aug 20;21:569. doi: 10.1186/s12864-020-06971-7 (PMC7440613; doi:10.1186/s12864-020-06971-7)
Supplement: Supplementary file 1 — Additional file 1: Figure S1. Maximum likelihood tree of the 66 genomes based on single-copy marker proteins. Phylogeny was inferred from the concatenation of 120 conserved amino-acid sequences by GTDB-Tk. Black circles in the middle of the branches represent Shimodaira-Hasegawa (SH) likelihood support values. Colour annotations represent the different clades and phyla. Sequences belonging to Cyanobacteria and Proteobacteria were used as outgroups. Names in bold indicate sequences generated in the present study. Scale bar represents amino acid substitutions per site. Table S1. Strains and growth media. Details on the preparation of the media and the cultivation conditions can be found in the References section. Table S2. Taxonomic assignment of flavobacterial strains sequenced in this study. Information on the 16S rRNA gene sequence of each strain, BLASTN best hits against nr/nt NCBI database and GTDB-Tk classification. Table S3. Pfam entries most strongly contributing to differentiating genomes from different Flavobacteriaceae clades. Pfam entries with the highest significant contribution (> 0.2%, p < 0.05) to the dissimilarity are shown. M, Marine; C, Capnocytophaga; T, Tenacibaculum-Polaribacter; F, Flavobacterium. Table S4. Number of BGCs, average gene counts and % of genes in BGCs per group. Table S5. Distribution of identified BGCs across different groups and Flavobacteriaceae clades. M, Marine; C, Capnocytophaga; F, Flavobacterium; T, Tenacibaculum-Polaribacter. Table S6. Cultivation conditions of indicator strains and antibiotics used in the antimicrobial activity tests. [file 12864_2020_6971_MOESM1_ESM.docx]

**Supplementary Information**

**Comparative genomic analysis of *Flavobacteriaceae*: insights into carbohydrate metabolism, gliding motility and secondary metabolite biosynthesis.**

Asimenia Gavriilidou, Johanna Gutleben, Dennis Versluis, Francesca Forgiarini, Mark W.J. van Passel, Colin J. Ingham, Hauke Smidt and Detmer Sipkema.

**Table of contents**

[**Figure S1** 3](#_Toc39528114)

[**Table S1** 4](#_Toc39528115)

[**Table S2** 5](#_Toc39528116)

[**Table S3** 5](#_Toc39528117)

[**Table S4** 8](#_Toc39528118)

[**Table S5** 8](#_Toc39528119)

[**Table S6** 11](#_Toc39528120)

**Figure S1.** **Maximum likelihood tree of the 66 genomes based on single-copy marker proteins.** Phylogeny was inferred from the concatenation of 120 conserved amino-acid sequences by GTDB-Tk. Black circles in the middle of the branches represent Shimodaira-Hasegawa (SH) likelihood support values. Colour annotations represent the different clades and phyla. Sequences belonging to *Cyanobacteria* and *Proteobacteria* were used as outgroups. Names in bold indicate sequences generated in the present study. Scale bar represents amino acid substitutions per site.

**Table S1. Strains and growth media.** Details on the preparation of the media and the cultivation conditions can be found in the References section.

| Strain ID | Isolation source | Growth Medium | References |
| --- | --- | --- | --- |
| DN50 | *A. aerophoba* | Mucin & Marine agar | Versluis et al. (2017) |
| DN105 | *A. aerophoba* | Marine agar | Versluis et al. (2017) |
| DN112 | *A. aerophoba* | Marine agar | Versluis et al. (2017) |
| Da_B9 | *D. avara* | Marine agar | unpublished |
| Aa_F7 | *A. aerophoba* | Mucin (50x) | Gutleben et al. (2020) |
| Aa_C5 | *A. aerophoba* | All amino acids (50x) | Gutleben et al. (2020) |
| Aa_D4 | *A. aerophoba* | All amino acids (50x) | Gutleben et al. (2020) |

**Table S2. Taxonomic assignment of flavobacterial strains sequenced in this study.** Information on the 16S rRNA gene sequence of each strain, BLASTN best hits against nr/nt NCBI database and GTDB-Tk classification.

| Strain ID | 16S rRNA gene length (bp) | Closest BLASTN hit (Accession number) | ID% | GTDB-Tk classification |
| --- | --- | --- | --- | --- |
| Aa_C5 | 1362 | *Eudoraea* *chungangensis* (NR_148299.1) | 99.7 | d__Bacteria;p__Bacteroidota;c__Bacteroidia;o__Flavobacteriales;f__Flavobacteriaceae;g__Eudoraea;s |
| Aa_D4 | 1349 | *Eudoraea* *chungangensis* (NR_148299.1) | 99.7 | d__Bacteria;p__Bacteroidota;c__Bacteroidia;o__Flavobacteriales;f__Flavobacteriaceae;g__Eudoraea;s |
| Aa_F7 | 1257 | *Flagellimonas* sp. (MF287795.1) | 97.9 | d__Bacteria;p__Bacteroidota;c__Bacteroidia;o__Flavobacteriales;f__Flavobacteriaceae;g__Flagellimonas;s |
| Da_B9 | 1510 | *Flavobacteriaceae* bacterium (MK956921.1) | 94.0 | d__Bacteria;p__Bacteroidota;c__Bacteroidia;o__Flavobacteriales;f__Flavobacteriaceae;g__;s |
| DN50 | 1121 | Uncultured organism clone (DQ395462.1) | 96.6 | d__Bacteria;p__Bacteroidota;c__Bacteroidia;o__Flavobacteriales;f__Flavobacteriaceae;g__GCA-2746415;s |
| DN105 | 1190 | *Flavobacteriaceae* bacterium (EU581699.1) | 98.8 | d__Bacteria;p__Bacteroidota;c__Bacteroidia;o__Flavobacteriales;f__Flavobacteriaceae;g__Flaviramulus_A;s |
| DN112 | 1391 | *Lacinutrix* sp. (KX398615.1) | 98.2 | d__Bacteria;p__Bacteroidota;c__Bacteroidia;o__Flavobacteriales;f__Flavobacteriaceae;g__;s |

**Table S3**. **Pfam entries most strongly contributing to differentiating genomes from different Flavobacteriaceae clades.** Pfam entries with the highest significant contribution (> 0.2%, p < 0.05) to the dissimilarity are shown. M, Marine; C, *Capnocytophaga*; T, *Tenacibaculum*-*Polaribacter*; F, *Flavobacterium*.

| Function name (Pfam ID) | Relative Abundance (%) | | | | Contribution (%) | | | p-values (< 0.05) | | |
| --- | --- | --- | --- | --- | --- | --- | --- | --- | --- | --- |
|  | M | C | T | F | M-C | M-T | M-F | M-C | M-T | M-F |
| CarboxypepD_reg-like domain (pfam13715) | 1.20 | 1.78 | 1.28 | 0.95 | 1.06 | 0.51 | 0.68 | 0.011 | 0.999 | 0.398 |
| TonB-dependent Receptor Plug Domain (pfam07715) | 1.08 | 1.65 | 1.16 | 0.97 | 0.93 | 0.64 | 0.57 | 0.01 | 0.984 | 0.731 |
| SusD family (pfam07980) | 0.30 | 0.81 | 0.26 | 0.17 | 0.90 | 0.36 | 0.41 | 0.002 | 1 | 0.616 |
| Starch-binding associating with outer membrane (pfam14322) | 0.28 | 0.78 | 0.24 | 0.17 | 0.71 | 0.38 | 0.43 | 0.002 | 1 | 0.591 |
| TonB dependent receptor (pfam00593) | 0.53 | 0.90 | 0.47 | 0.45 | 0.62 | 0.36 | 0.43 | 0.01 | 1 | 0.438 |
| Sigma-70, region 4 (pfam08281) | 0.53 | 0.62 | 0.52 | 0.26 | 0.42 | 0.23 | 0.39 | 0.04 | 0.999 | 0.027 |
| FecR protein (pfam04773) | 0.12 | 0.31 | 0.08 | 0.04 | 0.41 | 0.17 | 0.18 | 0.015 | 0.977 | 0.492 |
| Domain of unknown function (DUF4974) (pfam16344) | 0.12 | 0.30 | 0.08 | 0.05 | 0.40 | 0.17 | 0.19 | 0.016 | 0.978 | 0.469 |
| Glycosyl hydrolases family 43 (pfam04616) | 0.06 | 0.28 | 0.02 | 0.03 | 0.40 | 0.10 | 0.11 | 0.001 | 0.998 | 0.931 |
| Phage integrase family (pfam00589) | 0.19 | 0.32 | 0.19 | 0.27 | 0.33 | 0.15 | 0.21 | 0.015 | 0.999 | 0.112 |
| Outer membrane efflux protein (pfam02321) | 0.25 | 0.44 | 0.28 | 0.31 | 0.32 | 0.13 | 0.14 | 0.001 | 0.993 | 0.432 |
| Barrel-sandwich domain of CusB or HlyD membrane-fusion (pfam16576) | 0.20 | 0.37 | 0.22 | 0.29 | 0.31 | 0.16 | 0.16 | 0.001 | 0.676 | 0.252 |
| AcrB/AcrD/AcrF family (pfam00873) | 0.16 | 0.33 | 0.19 | 0.19 | 0.31 | 0.13 | 0.12 | 0.001 | 0.888 | 0.606 |
| ABC transporter (pfam00005) | 0.88 | 0.79 | 0.86 | 0.96 | 0.30 | 0.22 | 0.23 | 0.039 | 0.955 | 0.202 |
| Tetratricopeptide repeat (pfam13181) | 0.33 | 0.16 | 0.27 | 0.24 | 0.29 | 0.20 | 0.30 | 0.022 | 0.938 | 0.574 |
| Phage integrase SAM-like domain (pfam13102) | 0.09 | 0.23 | 0.09 | 0.06 | 0.28 | 0.15 | 0.09 | 0.003 | 0.685 | 0.996 |
| Integrase core domain (pfam00665) | 0.03 | 0.18 | 0.01 | 0.11 | 0.27 | 0.07 | 0.16 | 0.016 | 0.999 | 0.141 |
| Glycosyl hydrolases family 2, sugar binding domain (pfam02837) | 0.08 | 0.20 | 0.06 | 0.05 | 0.25 | 0.17 | 0.15 | 0.04 | 0.733 | 0.514 |
| Transposase DDE domain (pfam01609) | 0.01 | 0.15 | 0.00 | 0.15 | 0.25 | 0.02 | 0.23 | 0.048 | 0.999 | 0.027 |
| Glycosyl hydrolases family 2 (pfam00703) | 0.07 | 0.19 | 0.06 | 0.06 | 0.24 | 0.17 | 0.14 | 0.033 | 0.669 | 0.567 |
| IstB-like ATP binding protein (pfam01695) | 0.00 | 0.14 | 0.00 | 0.00 | 0.24 | 0.00 | 0.00 | 0.001 | 0.999 | 1 |
| Starch-binding associating with outer membrane (pfam12771) | 0.08 | 0.18 | 0.08 | 0.06 | 0.20 | 0.10 | 0.08 | 0.005 | 0.968 | 0.992 |
| Heavy-metal-associated domain (pfam00403) | 0.13 | 0.23 | 0.19 | 0.08 | 0.20 | 0.16 | 0.10 | 0.024 | 0.409 | 0.932 |
| C-terminal domain of CHU protein family (pfam13585) | 0.41 | 0.26 | 0.26 | 0.37 | 0.27 | 0.30 | 0.19 | 0.061 | 0 .045 | 0.461 |
| Response regulator receiver domain (pfam00072) | 1.11 | 1.01 | 1.08 | 1.04 | 0.46 | 0.39 | 0.52 | 0.529 | 0.995 | 0.037 |
| Sigma-70 region 2 (pfam04542) | 0.66 | 0.71 | 0.60 | 0.32 | 0.45 | 0.23 | 0.43 | 0.063 | 1 | 0.003 |
| Helix-turn-helix (pfam01381) | 0.18 | 0.27 | 0.18 | 0.33 | 0.21 | 0.16 | 0.30 | 0.386 | 0.994 | 0.005 |
| Enoyl-(Acyl carrier protein) reductase (pfam13561) | 0.33 | 0.34 | 0.27 | 0.19 | 0.20 | 0.21 | 0.26 | 0.834 | 0.938 | 0.024 |
| Sodium:solute symporter family (pfam00474) | 0.18 | 0.20 | 0.20 | 0.05 | 0.11 | 0.13 | 0.20 | 0.995 | 0.88 | 0.001 |

**Table S4**. **Number of BGCs, average gene counts and % of genes in BGCs per group.**

| Groups | Total BGCs | Average BGCs | Average genes | % genes in BGCs |
| --- | --- | --- | --- | --- |
| *Flavobacteriaceae* (n = 56) | 197 | 4 | 4447 | 0.1 |
| Marine (n = 41) | 151 | 4 | 3668 | 0.1 |
| *Capnocytophaga* (n = 5) | 15 | 3 | 3591 | 0.1 |
| *Flavobacterium* (n = 5) | 20 | 4 | 3200 | 0.1 |
| *Tenacibaculum*-*Polaribacter* (n = 5) | 11 | 2 | 3055 | 0.1 |
| *Cyanobacteria* (n = 5) | 33 | 7 | 3397 | 0.3 |
| *Proteobacteria* (n = 5) | 20 | 4 | 3565 | 0.1 |

**Table S5**. **Distribution of identified BGCs across different groups and Flavobacteriaceae clades.** M, Marine; C, *Capnocytophaga*; F, *Flavobacterium*; T, *Tenacibaculum*-*Polaribacter*.

| Gene cluster type (antiSMASH) | *Flavobacteriaceae* | | | | | | | | *Cyanobacteria* | | *Proteobacteria* | | |
| --- | --- | --- | --- | --- | --- | --- | --- | --- | --- | --- | --- | --- | --- |
|  | M | | C | | F | | T | |  |  |  |  |  |
|  | Total | % | Total | % | Total | % | Total | % | Total | % | Total | % |  |
| Aryl polyene | 7 | 4.6 | 0 | 0.0 | 3 | 15.0 | 1 | 9.1 | 3 | 9.09 | 1 | 5.0 |  |
| Aryl polyene-Resorcinol | 7 | 4.6 | 0 | 0.0 | 3 | 15.0 | 0 | 0.0 | 0 | 0.00 | 0 | 0.0 |  |
| Aryl polyene-Resorcinol-T3PKS | 1 | 0.7 | 0 | 0.0 | 0 | 0.0 | 0 | 0.0 | 0 | 0.00 | 0 | 0.0 |  |
| Bacteriocin | 5 | 3.3 | 1 | 6.7 | 1 | 5.0 | 0 | 0.0 | 7 | 21.21 | 4 | 20.0 |  |
| Bacteriocin-Lanthipeptide | 0 | 0.0 | 0 | 0.0 | 0 | 0.0 | 0 | 0.0 | 1 | 3.03 | 0 | 0.0 |  |
| Betalactone | 0 | 0.0 | 0 | 0.0 | 1 | 5.0 | 0 | 0.0 | 0 | 0.00 | 2 | 10.0 |  |
| Ectoine | 0 | 0.0 | 0 | 0.0 | 0 | 0.0 | 0 | 0.0 | 0 | 0.00 | 1 | 5.0 |  |
| Hserlactone | 1 | 0.7 | 0 | 0.0 | 0 | 0.0 | 0 | 0.0 | 0 | 0.00 | 7 | 35.0 |  |
| Ladderane | 0 | 0.0 | 1 | 6.7 | 0 | 0.0 | 0 | 0.0 | 0 | 0.00 | 0 | 0.0 |  |
| Lanthipeptide | 9 | 6.0 | 2 | 13.3 | 0 | 0.0 | 1 | 9.1 | 0 | 0.00 | 0 | 0.0 |  |
| LAP-Bacteriocin-Cyanobactin | 0 | 0.0 | 0 | 0.0 | 0 | 0.0 | 0 | 0.0 | 1 | 3.03 | 0 | 0.0 |  |
| Lassopeptide | 0 | 0.0 | 0 | 0.0 | 0 | 0.0 | 0 | 0.0 | 0 | 0.00 | 1 | 5.0 |  |
| Linaridin | 2 | 1.3 | 0 | 0.0 | 0 | 0.0 | 0 | 0.0 | 0 | 0.00 | 0 | 0.0 |  |
| Microviridin | 0 | 0.0 | 1 | 6.7 | 0 | 0.0 | 0 | 0.0 | 0 | 0.00 | 0 | 0.0 |  |
| NRPS | 12 | 7.9 | 0 | 0.0 | 1 | 5.0 | 0 | 0.0 | 5 | 15.15 | 0 | 0.0 |  |
| NRPS-like | 2 | 1.3 | 2 | 13.3 | 0 | 0.0 | 0 | 0.0 | 3 | 9.09 | 0 | 0.0 |  |
| NRPS-like-T1PKS | 0 | 0.0 | 0 | 0.0 | 0 | 0.0 | 0 | 0.0 | 0 | 0.00 | 1 | 5.0 |  |
| NRPS-T1PKS | 8 | 5.3 | 0 | 0.0 | 0 | 0.0 | 0 | 0.0 | 2 | 6.06 | 0 | 0.0 |  |
| Resorcinol | 2 | 1.3 | 1 | 6.7 | 0 | 0.0 | 0 | 0.0 | 0 | 0.00 | 0 | 0.0 |  |
| Siderophore | 0 | 0.0 | 0 | 0.0 | 3 | 15.0 | 1 | 9.1 | 0 | 0.00 | 1 | 5.0 |  |
| T1PKS | 1 | 0.7 | 1 | 6.7 | 0 | 0.0 | 0 | 0.0 | 1 | 3.03 | 0 | 0.0 |  |
| T1PKS-PUFA-hg1E-KS | 1 | 0.7 | 0 | 0.0 | 0 | 0.0 | 0 | 0.0 | 0 | 0.00 | 0 | 0.0 |  |
| T3PKS | 16 | 10.6 | 0 | 0.0 | 1 | 5.0 | 1 | 9.1 | 1 | 3.03 | 0 | 0.0 |  |
| T3PKS-Aryl polyene | 8 | 5.3 | 0 | 0.0 | 0 | 0.0 | 1 | 9.1 | 0 | 0.00 | 0 | 0.0 |  |
| Terpene | 67 | 44.4 | 6 | 40.0 | 7 | 35.0 | 6 | 54.5 | 8 | 24.24 | 2 | 10.0 |  |
| Terpene-Bacteriocin | 0 | 0.0 | 0 | 0.0 | 0 | 0.0 | 0 | 0.0 | 1 | 3.03 | 0 | 0.0 |  |
| TransAT-PKS-like, TransAT-PKS, PKS-like | 1 | 0.7 | 0 | 0.0 | 0 | 0.0 | 0 | 0.0 | 0 | 0.00 | 0 | 0.0 |  |
| TransAT-PKS-NRPS | 1 | 0.7 | 0 | 0.0 | 0 | 0.0 | 0 | 0.0 | 0 | 0.00 | 0 | 0.0 |  |

**Table S6**. **Cultivation conditions of indicator strains and antibiotics used in the antimicrobial activity tests.**

| Reference strains | Temperature (°C) | Media | Antibiotics (Positive control) |
| --- | --- | --- | --- |
| *Bacillus subtilis* (DSM 402) | 30 | Nutrient broth | Tetracycline |
| *Staphylococcus simulans* (DSM 20037) | 37 | Tryptic Soy broth (TSB) | Vancomycin |
| *Escherichia coli* (MIB, WUR) | 37 | Luria-Bertani broth (LB) | Tetracycline |
| *Aeromonas salmonicida* (DSM 19634) | 30 | Nutrient broth | Vancomycin |
| *Saprolegnia parasitica* (CBS 223.65) | 20 | Potato Dextrose agar (PDA) | Delvocid |
| *Candida oleophila* (DSM 70763) | 25 | Yeast Malt Broth (YMB) | Nystatin |
