# Supplementary material for: Comparative genomic analysis of Flavobacteriaceae: insights into carbohydrate metabolism, gliding motility and secondary metabolite biosynthesis
Source: BMC Genomics. 2020 Aug 20;21:569. doi: 10.1186/s12864-020-06971-7 (PMC7440613; doi:10.1186/s12864-020-06971-7)
Supplement: Supplementary file 2 — Additional file 2:. Publicly available genomes used in this study. [file 12864_2020_6971_MOESM2_ESM.pdf]

Additional File 2. Publicly available genomes used in this study (Source: https://gold.jgi.doe.gov/).

IMG Genome Name / Sample Name	IMG Genome ID	Sequencing Status	Genome size (bp)	Gene Count	16SrRNA GenBank accession number	Isolation source
Algibacter wandonensis CECT 8301	2772190795	Draft	4,707,503	4004	KC987358	sediment
Alteromonas macleodii ATCC 27126	2554235747	Finished	4,653,851	3941	CP003841	seawater
Anabaena sp. PCC 7108	2506485002	Permanent Draft	5,886,741	5227	AJWF01000009	seawater
Arenibacter echinorum DSM 23522	2593339296	Permanent Draft	5,193,588	4393	EF536748	sea urchin
Bizionia echini DSM 23925	2622736504	Draft	3,314,721	3072	FJ716799	sea urchin
Candidatus Pelagibacter ubique SAR11 HTCC1062	637000058	Finished	1,308,759	1394	CP000084	seawater
Cellulophaga fucicola DSM 24786	2595699004	Draft	3,922,041	3495	AJ005973	algae
Cellulophaga lytica LIM-21, DSM 7489	649633032	Finished	3,765,936	3358	AB517706	sediment
Cellulophaga tyrosinoxydans DSM 21164	2595698249	Permanent Draft	3,555,791	3209	EU443205	seawater
Crocosphaera watsonii WH 8501	2623620439	Draft	6,291,599	6836	AADV02000003	seawater
Dokdonia pacifica DSM 25597	2724679821	Draft	5,520,745	4968	KP862606	seawater
Dokdonia sp. PRO95	2597489917	Finished	3,305,093	3038	FJ627052	seawater
Eudoraea adriatica DSM 19308	2522572201	Permanent Draft	3,906,474	3579	AM745437	seawater
Flavobacterium branchiophilum FL-15	2561511155	Finished	3,563,292	2925	FQ859183	fish pathogen
Flavobacterium columnare ATCC 49512	2511231122	Finished	3,162,865	2735	CP003222	fish pathogen
Flavobacterium indicum GPTSA100-9	2540341066	Finished	2,993,089	2738	HE774682	spring water
Flavobacterium johnsoniae UW101, ATCC 17061	644736369	Finished	6,096,872	5099	CP000685	soil/freshwater
Flavobacterium psychrophilum JIP02/86	640753027	Finished	2,861,988	2505	AM398681	fish pathogen
Formosa agariphila KMM 3901	2585427664	Permanent Draft	4,228,350	3630	AY187688	algae

Formosa spongicola DSM 22637	2595698203	Permanent Draft	3,155,334	2961	FJ348469	sponge
Gaetbulibacter saemankumensis DSM 17032	2524614665	Permanent Draft	3,089,149	2746	AY883937	sediment
Galbibacter marinus ck-I2-15	2519899576	Permanent Draft	3,572,447	3138	EU928746	sediment
Idiomarina loihiensis GSL 199	2554235415	Finished	2,839,759	2717	CP005964	seawater
Imtechella halotolerans K1	2534681666	Permanent Draft	3,086,951	2734	FR774044	brackish water
Joostella marina DSM 19592	2509276026	Permanent Draft	4,508,243	4004	EF660761	seawater
Kordia algicida OT-1	641380434	Permanent Draft	5,019,836	4584	AY195836	seawater
Kordia periserrulae DSM 25731	2734482288	Draft	4,725,576	4128	GU233518	polychaete
Kriegella aquimaris DSM 19886	2622736525	Draft	6,057,242	5014	AB084262	seawater
Lacinutrix sp. Hel_I _90	2582581868	Permanent Draft	3,819,763	3506	JX854138	seawater
Lacinutrix venerupis DOK2-8	2751185745	Draft	3,192,399	2890	CP019352	seawater
Lutibacter maritimus DSM 24450	2622736611	Draft	3,484,703	3147	FJ598048	sediment
Maribacter arcticus DSM 23546	2595698209	Permanent Draft	4,211,145	3760	AY771762	sediment
Maribacter polysiphoniae DSM 23514	2595698208	Permanent Draft	5,129,962	4489	AM497875	algae
Maribacter spongiicola DSM 25233	2731957517	Draft	4,455,565	3894	JX050191	sponge
Maribacter vaceletii DSM 25230	2734482098	Draft	3,889,815	3392	JX050190	sponge
Muricauda antarctica DSM 26351	2619619048	Draft	4,482,831	4102	JN166984	seawater
Muricauda pacifica DSM 25027	2731639122	Draft	4,376,054	4153	JN118551	seawater
Muriicola jejuensis DSM 21206	2724679820	Draft	3,299,848	3040	EU443206	seawater
Nonlabens spongiae JCM 13191	2751185769	Draft	3,393,335	3097	DQ064789	sponge
Nonlabens ulvanivorans DSM 22727	2593339289	Permanent Draft	3,177,440	2918	GU902979	algae
Polaribacter haliotis RA4-7	2788500510	Draft	3,780,569	3361	KX450477	abalone
Polaribacter sp. MED152	638341218	Finished	2,967,100	2723	CP004349	seawater

Prochlorococcus marinus MIT9515	640069324	Finished	1,704,176	1968	CP000552	seawater
Psychroserpens sp. Hel_I_66	2585427602	Permanent Draft	3,842,990	3475	JUGU01000001	seawater
Pustulibacterium marinum CGMCC 1.12333	2663762752	Draft	4,209,902	3796	FPBK01000031	seawater
Robiginitalea myxolifaciens DSM 21019	2636416043	Draft	3,206,962	2897	AB270585	sediment
Roseobacter litoralis Och 149	2510065042	Finished	4,745,450	4577	CP002623	macroalgae
Ruegeria pomeroyi DSS-3	637000267	Finished	4,601,053	4355	CP000031	seawater
Sinomicrobium oceani CGMCC 1.12145	2596583572	Draft	5,026,272	4281	FPJE01000054	sediment
Synechococcus sp. WH 8016	2507262052	Finished	2,706,690	3046	AGIK01000004	seawater
Tamlana sedimentorum JCM 19808	2636415452	Draft	3,961,353	3504	AB894238	sediment
Tenacibaculum adriaticum DSM 18961	2756170228	Draft	3,211,939	2987	AM412314	bryozoan
Tenacibaculum mesophilum DSM 13764	2695420951	Draft	3,286,619	3059	AB032501	sponge
Trichodesmium erythraeum IMS101	637000329	Finished	7,750,108	5156	CP000393	seawater
Winogradskyella arenosi CECT 7958	2770939486	Draft	3,675,157	3253	AB438962	sediment
Winogradskyella eximia CECT 7946	2770939573	Draft	4,242,526	3742	AY521225	algae
Winogradskyella jejuensis DSM 25330	2695420983	Draft	3,033,897	2864	JF820844	algae
Winogradskyella sp. J14-2	2751185740	Draft	3,349,669	3049	CP019388	seawater
Zobellia galactanivorans DsijT	2619619092	Draft	5,521,712	4563	AF208293	algae
